# Using genomic epidemiology of SARS-CoV-2 to support contact tracing and public health surveillance in rural Humboldt County, California

**DOI:** 10.1186/s12889-022-12790-0

**Published:** 2022-03-07

**Authors:** Gunnar Stoddard, Allison Black, Patrick Ayscue, Dan Lu, Jack Kamm, Karan Bhatt, Lienna Chan, Amy L Kistler, Joshua Batson, Angela Detweiler, Michelle Tan, Norma Neff, Joseph L DeRisi, Jeremy Corrigan

**Affiliations:** 1 Humboldt County Department of Health and Human Services - Public Health, CA Eureka, USA; 2 Chan Zuckerberg Initiative, CA San Francisco, USA; 3 Chan Zuckerberg Biohub, CA San Francisco, USA; 4 Department of Biochemistry and Biophysics, University of California San Francisco, San Francisco, CA USA; 5 Humboldt County Public Health Laboratory, CA Eureka, USA

**Keywords:** SARS-CoV-2, Genomic epidemiology, Public health response

## Abstract

**Background:**

During the COVID-19 pandemic within the United States, much of the responsibility for diagnostic testing and epidemiologic response has relied on the action of county-level departments of public health. Here we describe the integration of genomic surveillance into epidemiologic response within Humboldt County, a rural county in northwest California.

**Methods:**

Through a collaborative effort, 853 whole SARS-CoV-2 genomes were generated, representing ~58% of the 1,449 SARS-CoV-2-positive cases detected in Humboldt County as of March 12, 2021. Phylogenetic analysis of these data was used to develop a comprehensive understanding of SARS-CoV-2 introductions to the county and to support contact tracing and epidemiologic investigations of all large outbreaks in the county.

**Results:**

In the case of an outbreak on a commercial farm, viral genomic data were used to validate reported epidemiologic links and link additional cases within the community who did not report a farm exposure to the outbreak. During a separate outbreak within a skilled nursing facility, genomic surveillance data were used to rule out the putative index case, detect the emergence of an independent Spike:N501Y substitution, and verify that the outbreak had been brought under control.

**Conclusions:**

These use cases demonstrate how developing genomic surveillance capacity within local public health departments can support timely and responsive deployment of genomic epidemiology for surveillance and outbreak response based on local needs and priorities.

**Supplementary Information:**

The online version contains supplementary material available at 10.1186/s12889-022-12790-0.

## Introduction

In early December 2019 a novel human coronavirus, now known as SARS-CoV-2, emerged in Wuhan, China. During January and February 2020, travel-associated cases of COVID-19, the disease caused by SARS-CoV-2, surfaced within the United States. By the end of February 2020 the United States reported instances of community transmission [1]. Over the following year, the scope of the resulting pandemic has become clear. There have been greater than 33 million reported cases nationally, and 3.7 million just in California as of this writing (May 2021).

Within the United States, frontline public health activities typically fall under the jurisdictional authority of states, counties, or smaller administrative units. County-level public health departments are routinely responsible for performing diagnostic testing, mounting epidemiologic responses, and reporting notifiable illnesses or conditions to higher level jurisdictional authorities. Thus, much of the responsibility for detecting and preventing COVID-19 transmission has relied on the action of county-level departments of public health. Despite a remit to conduct similar public health activities, the capacities and resources available to departments of public health vary greatly across the United States, which has resulted in an uneven response to the pandemic.

When compared to other counties in California, the unique features of Humboldt County present particular challenges with respect to pandemic response, especially for COVID-19. Humboldt County is a rural county in northwest California encompassing 2.3 million acres, 80% of which is forestland, protected redwoods and recreation areas, serviced by a single small regional airport. The county is a healthcare and behavioral healthcare provider shortage area, meaning that many of the county’s 135,727 residents must travel long distances for access to healthcare services, and the largest population center (Eureka-Arcata) may be isolated due to landslides or other natural disasters. Many Humboldt residents are uninsured and more than one in five individuals live at or below the federal poverty line. The largest minority populations are Native American and Latino, both at high risk for serious COVID-19 disease, and the area experiences notable health disparities and overall poorer health outcomes when compared to state and national data [2]. Furthermore, pandemic preparedness has been hampered by a dearth of laboratory facilities in the region. The Humboldt County Public Health Laboratory (HCPHL) was the only local testing laboratory for SARS-CoV-2 early in the pandemic, with local hospitals and clinics adding testing options subsequently.

Despite these challenges, Humboldt County has used genomic sequencing of SARS-CoV-2 as a key component in the arsenal of epidemiological tools used to monitor, track, and control spread of the virus. Epidemiological analyses of viral sequence data have played critical roles in our understanding of SARS-CoV-2 epidemiology, and have been used to detect cryptic transmission of SARS-CoV-2 [3, 4], identify multiple, independent introductions of SARS-CoV-2 [4–6], characterize SARS-CoV-2 transmission patterns domestically within the United States [7], and calculate the increased transmissibility of newly detected variants [8], including here in California [9]. Despite their value, the technical complexity of genomic surveillance systems has often limited their accessibility outside of higher resource urban settings, including at the county-level within the United States. In this paper we describe how, within a rural county locale, we improved diagnostic testing capacity and implemented dense genomic surveillance of SARS-CoV-2. We then discuss how findings from genomic surveillance data supported public health surveillance and guided epidemiologic response efforts in Humboldt County, with specific attention on two outbreaks, one in a farm setting, and the other in the context of a skilled nursing facility. Humboldt County’s pandemic response can offer a roadmap for other local jurisdictions outside urban centers evaluating how to incorporate genomic epidemiology into their response efforts.

## Methods

### Collection of SARS-CoV-2 diagnostic specimens

Samples were collected from a variety of submitters throughout Humboldt County including local hospitals, clinics, skilled nursing facilities (SNFs), assisted living facilities (ALFs), other congregate living facilities such as behavioral health centers, the county jail, local college dormitories and local health centers. Samples were typically collected upon clinical suspicion of SARS-CoV-2 infection or as part of case investigations conducted by the Humboldt County Department of Health and Human Services - Public Health (DHHS-PH), which performed targeted prospective surveillance in populations of concern such as SNFs and ALFs. While some SNFs were subject to routine surveillance testing to identify possible outbreaks early on, most samples were collected from symptomatic individuals and thus represent a skewed sample of all infections that likely occurred within Humboldt County.

Throughout the pandemic the sample type evolved. Initially, samples were collected using dual or combined nasopharyngeal/oropharyngeal swabs. More recently, nasopharyngeal swabs and observed self-collected nasal swabs were the primary sample types that were received and tested. Additionally, swabs in both viral transport media (VTM) and saline transport media were validated for testing. Samples were collected and transported to the lab as soon as possible and held at 4 degrees Celsius for up to 72 h, or frozen for up to 7 days if sample processing was delayed.

### Logistics of SARS-CoV-2 testing in Humboldt County

Early in the pandemic the majority of specimens collected by health care providers within Humboldt County requiring SARS-CoV-2 testing were handled by the HCPHL. A prioritization system was established to ensure that symptomatic samples and high risk populations were tested locally, which improved result turnaround times. Low priority samples, such as those collected to provide travel clearance, pre-operative screening, and general population surveillance, were deferred to commercial laboratories such as Quest and LabCorp. SARS-CoV-2 testing of hospital in-patients was mostly conducted by the hospital. Additionally, some specimens were collected and tested by OptumServe through a partnership with the Californian government to increase testing capacity.

The OptumServe testing site served as a community surveillance site and began operating in April 2020. The site was initially a walk-in sample collection service that operated 5 days per week, collecting approximately 120 samples per day. Optum sample collection capacity increased over time, expanding to two collection teams which increased capacity up to 319 samples per day. Additionally, an Optum mobile team provided capacity to collect up to 561 samples per week. All samples collected by Optum were tested at the California Department of Public Health’s Valencia Branch Laboratory. None of these samples were sequenced as part of this project. Finally, Humboldt County, Del Norte County and United Indian Health Services formed a regional COVID testing task force to coordinate testing strategy and resources for the region. Analysis by the task force led to the establishment of the North Coast Testing Partnership (NCTP) which began performing diagnostic testing in December 2020. The NCTP lab tested between 100 and 200 samples 5 days a week.

### Viral RNA extraction

Throughout the pandemic the HCPHL used a variety of extraction platforms. RNA extractions were initially performed manually using the QIAamp Viral RNA mini kit (Qiagen) following manufacturer instructions. To improve throughput, automated RNA extraction was implemented on the MagNAPure Compact (Roche), eluting the extracted sample in 100 µls of elution buffer. Additionally, some extractions were either performed on the Qiagen EZ1 Advanced, eluting samples in 120 µls of elution buffer, or on the KingFisher™ Flex Purification System and the MagMAX™ Viral/Pathogen II Nucleic Acid Isolation Kit (ThermoFisher), which eluted the sample in 100 µls of elution buffer.

### SARS-CoV-2 diagnostic testing

At the beginning of the pandemic, HCPHL used the CDC 2019-Novel Coronavirus (2019-nCoV) Real-Time RT-PCR Diagnostic Panel. This assay is a singleplex assay which only allows processing of 10 samples at a time. In August of 2020 we began using the TaqPath COVID-19 Combo Kit (ThermoFisher), which allowed multiplexing of 93 samples per run. In preparation for Influenza season, HCPHL transitioned to the CDC Influenza SARS-CoV-2 (Flu SC2) Multiplex Assay that identifies SARS-CoV-2, Influenza A, and Influenza B simultaneously and tests 93 samples per run. Recent diagnostic testing primarily used the CDC Flu SC2 Multiplex Assay. To accommodate rapid turnaround of high-priority specimens, the HCPHL also validated the Xpert Xpress SARS-CoV-2 assay using the GeneXpert testing system (CEPHEID). The lab upgraded the GeneXpert testing system from 4 modules to 16 modules to increase capacity and reduce turn-around time. Additionally, the Xpert SARS-CoV-2/Flu/RSV 4-plex assay (CEPHEID) was validated and used during the influenza season. All PCR assays were used according to manufacturer instructions and instructions for use (IFU) without deviation.

### Selection of samples for sequencing

Convenience samples of diagnostic specimens were sent periodically for whole genome sequencing at the Chan Zuckerberg Biohub in San Francisco. Viral load was used as the primary criteria for choosing samples to sequence given that samples with higher viral loads are more likely to yield high quality whole genome sequences. All samples tested by HCPHL with RT-PCR cycle threshold (Ct) values of less than 31 were sent for sequencing. Additional samples of epidemiologic interest, such as specimens associated with specific case investigations, were also included. Eluted RNA was aliquoted into 96-well plates and sent to the Biohub in batches ranging from 40 to 96 samples. Metadata were compiled by communicable disease investigators to assist in post-sequencing analysis and case investigations.

### Sequencing of SARS-CoV-2 whole genomes

Extracted total nucleic acid was diluted based on average SARS-CoV-2 N and E gene cycle threshold (Ct) values; samples with a Ct range 12-15 were diluted 1:100, 15-18 1:10 and >18 no dilution. For high throughput scaling, library preparation reaction volumes and dilutions were miniaturized utilizing acoustic liquid handling (https://protocols.io/view/artic-neb-tagmentation-protocol-high-throughput-wh-bt66nrhe). Briefly, 3 µl of total nucleic acid was used as input for a randomly primed cDNA synthesis reaction. This cDNA served as input for 30 cycles of amplification with ARTIC V3 primers (primer sequences available at https://github.com/artic-network/artic-ncov2019), and was then diluted 1:100 before tagmentation. Adaptor tagmentation was performed using homebrew Tn5, and 8 cycles of index PCR was performed using unique dual barcode Nextera indices. Final libraries were pooled at equal volumes and cleaned at 0.7x (SPRI: Sample) using SPRIselect beads. The library was sequenced on the Illumina Novaseq SP platform in a paired-end 2 × 150 cycle run.

A subset of initial samples were library prepared using the Tailed Amplicon Sequencing V.2 with only primer pairs 71-84 of the ARTIC V3 primers to tile all of the S gene. Final libraries were sequenced by paired-end 2 × 150 bp sequencing on an Illumina NovaSeq platform.

### SARS-CoV-2 consensus genome generation

SARS-CoV-2 consensus genomes were generated from raw FASTQ files using the same bioinformatic processes and parameters as defined in [9]. Base calls were only made at sites with at least 10x high quality read depth, and unambiguous calls were only made if 90% or more of reads at a site specified one particular nucleotide. Viral genomes were uploaded to GISAID [10], and to NCBI Genbank [11]) if they had at least 27,500nt (92%) genome coverage with unambiguous base calls and less than 50 non-N ambiguous base calls.

### Phylogenetic dataset collation and analysis

To provide phylogenetic context for viral genomes sampled from Humboldt County, SARS-CoV-2 genome sequences representing global viral diversity were downloaded from GISAID [10, 12]. All phylogenetic analyses to support public health genomic surveillance and to generate figures for this paper were conducted using the Nextstrain tool suite, described in [13]. We used the *Augur* pipeline to align the sequences using nextalign v0.1.6, build a maximum likelihood phylogenetic tree with IQTREE v2.0.3 [14], and temporally resolve the tree using TreeTime v0.8.1 [15]. Final trees, which *Augur* also annotates with nucleotide and amino acid changes across the tree, were exported for visualization in *Auspice*, a web-based application which allows interactive exploration of the phylogenetic trees. To label trees with additional demographic and exposure data describing sequenced cases, we utilized the metadata “drag-and-drop” feature within *Auspice*, which allowed tips in the tree to be colored according to additional data fields specified in a tab-delimited file.

Given the wealth of data available from GISAID, viral genomes collected from regions other than Humboldt County were subsampled according to the following scheme. The 853 samples collected from Humboldt County were considered the focal samples. Given the sheer scale of whole genome sequences available in GISAID, sampling non-Humboldt sequences at random was unlikely to provide proper contextualization of where lineages sampled from Humboldt County circulated prior to migration into the county. Rather, subsampling was designed to maintain spatiotemporal diversity while enriching for contextual genome sequences that were genetically similar to Humboldt County focal samples. To do so, we sampled 50 sequences per month from all other Californian counties that were the least genetically diverged from Humboldt County sequences. Genome sequences from other states in the US were sampled at a rate of 50 genomes per month, again while enriching for sequences that were genetically more similar to sequences sampled from Humboldt County. Finally, to ensure proper rooting and global clade structure of the tree, 5 sequences were sampled per month from each of 6 global regions (Africa, South America, North America (excluding the US), Europe, Asia, and Oceania). The workflow describing the subsampling scheme can be found at https://github.com/alliblk/ncov-humboldt. Genomes used in the analysis had complete date information specifying the year, month and day of sample collection, and had at least 90% genome coverage with non-ambiguous base calls.

### Inference of introduction events to Humboldt County

The geographic migration history between locations was inferred across the tree using the discrete trait analysis method within TreeTime [15]. Under the phylogeographic model, inferred ancestral viruses (internal nodes within the tree) are annotated with the set of geographic areas where they may possibly have circulated and the probabilities of those states. We defined a migration event into Humboldt County as occurring if a parent node was inferred to circulate with greater than 50% probability anywhere outside of Humboldt County, and the child node was inferred with greater than 50% probability to circulate within Humboldt County. Migration events were considered to seed a discrete introduction into Humboldt County if viruses sampled from individuals residing within Humboldt County were descended from the internal node inferred to circulate within Humboldt County. Because discrete trait phylogeographic models treat sampling intensity as information about the underlying pathogen population size [16], a phylogeographic analysis with a highly overrepresented deme, such as we have performed here, will bias the reconstruction to favor earlier introductions and longer circulation times. Given this known source of bias, we consciously did not analyze source-sink dynamics or describe transmission directionality between Humboldt County and other geographic areas.

## Results

### Response to SARS-CoV-2 in Humboldt County

**Fig. 1:**
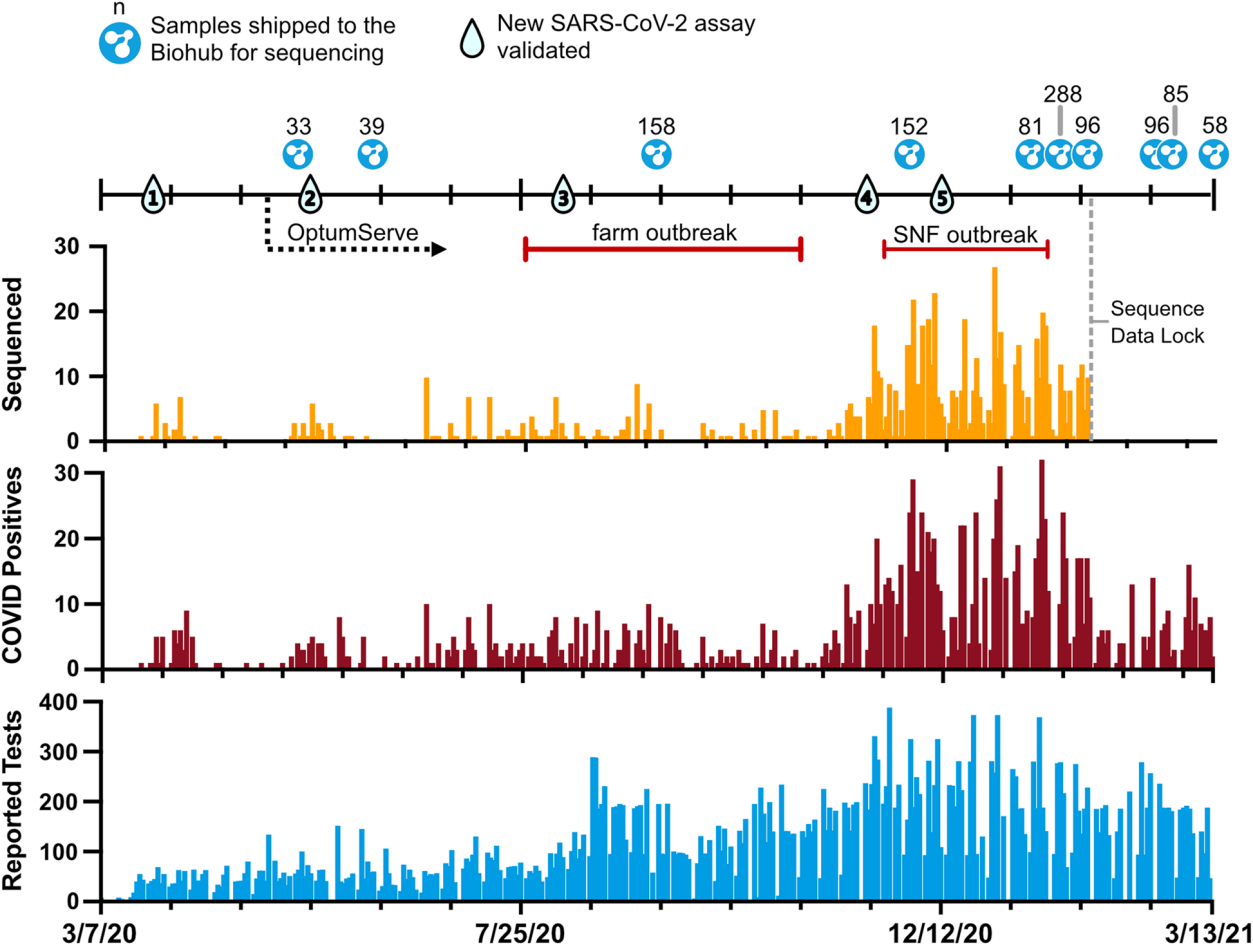
Overview and evolution of testing and sequencing for SARS-CoV-2 in Humboldt County. The blue bar chart indicates the number of tests performed in Humboldt County by the HCPHL by day since the start of testing through to the time of writing on March 13, 2021. The maroon bar chart indicates the number of qPCR-positive SARS-CoV-2 cases detected in Humboldt County over the same time period. The orange bar chart indicates the number of viral consensus genome sequences generated from diagnostic specimens up until the end of January 2021. Major changes to SARS-CoV-2 testing infrastructure are indicated with numbered droplet icons. These correspond to the following changes in SARS-CoV-2 testing infrastructure over time: 1: Switched from manual RNA extractions to automated extractions with Qiagen EZ1. 2: Validated the GeneXpert Xpress SARS-CoV-2 testing assay. 3: Switched from singleplex to TaqPath multiplex SARS-CoV-2 assay. 4: Switched to CDC SARS-CoV-2 multiplex assay using the KingFisher Flex. 5: Switched to multi-pathogen testing using GeneXpert Xpress SARS-CoV-2/Influenza/RSV 4-plex assay. Shipments for viral genome sequencing are indicated with the CZ Biohub logo

Beginning in February 2020, the HCPHL sent COVID-19 testing requests to the Centers for Disease Control and Prevention in Atlanta, Georgia. The first positive case of SARS-CoV-2 in Humboldt County occurred on February 21, 2020. Subsequently, HCPHL started onsite diagnostic testing via quantitative Polymerase Chain Reaction (qPCR) on March 7, 2020. The first case detected onsite by HCPHL occurred on March 19, 2020. The HCPHL capacity and testing strategy evolved over the pandemic, scaling from 10 samples per day at the start of the pandemic to 350 samples per day. By March 12, 2021, the laboratory had screened 35,499 possible cases and detected 1,449 (4.1%) PCR-confirmed SARS-CoV-2 infections (Fig. 1). During this time period, two major outbreaks occurred, as described below.

Like many county health laboratories, HCPHL lacked the resources and infrastructure to conduct whole genome sequencing of SARS-CoV-2 to support surveillance and outbreak response efforts. However, HCPHL established a partnership with the Chan Zuckerberg Biohub’s COVID Tracker Project, a project dedicated to whole genome sequencing and genomic epidemiologic interpretation in support of public health departments throughout California. The HCPHL sought sequencing support initially to assist with case investigations and to monitor for possible viral evolutionary changes that could affect diagnostic assays. The partnership was initiated in May, 2020 and enabled Humboldt County to perform genomic surveillance for SARS-CoV-2 throughout the majority of the pandemic, including monitoring for variants of concern later on. A total of 1,086 SARS-CoV-2 positive specimens were sent for sequencing during the time period described (Fig. 1).

### Most introductions of SARS-CoV-2 to Humboldt County appear self-limiting or highly-contained

The first sequenced genome from Humboldt County resulted from a sample collected on March 19, 2020. Between then and the sequence data lock for this manuscript on January 28, 2021, 853 viral whole genome sequences greater than 27,000nt in length were generated from the 1,086 samples sent to CZ Biohub (78.5%). We conducted a phylogeographic analysis of these 853 sequences alongside 1800 contextual SARS-CoV-2 whole genomes, primarily from other counties in California (n=1527), and including sequences from other global regions as well (see Methods for a description of the subsampling scheme).

**Fig. 2:**
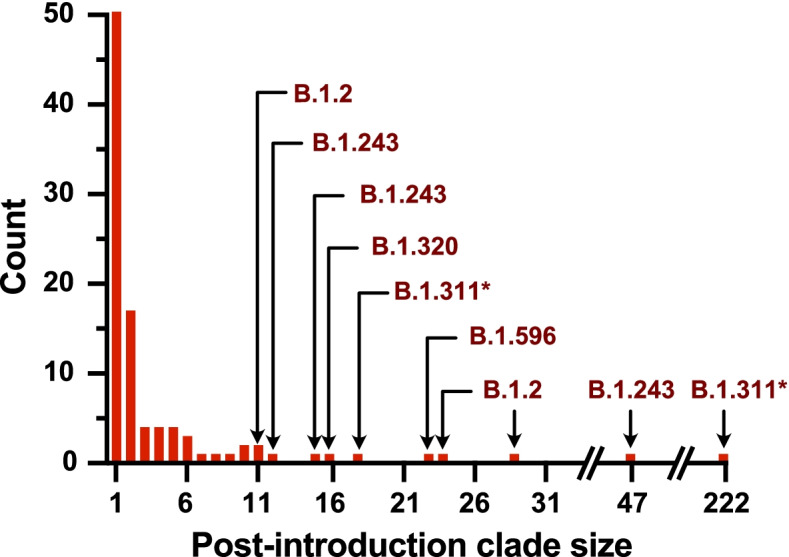
Histogram indicating the number of sequenced viruses grouping within distinct lineages introduced to Humboldt County over the course of the pandemic. For those introductions that resulted in greater than 10 post-introduction events, the Pango lineage is indicated. While the majority of introductions were limited to themselves, the largest post-introduction clade size was 222 events. The introduction resulting in 47 sequenced viruses contains the farm outbreak clade, and the introduction resulting in 222 sequenced viruses contains the SNF outbreak clade. *The parental B.1.311 lineage in this case gave rise to additional de novo mutations in Spike in some downstream members of this clade, including N501Y, and T95I

From our analysis we inferred that there were at least 100 discrete introductions of SARS-CoV-2 into Humboldt County (Supplemental Figs. 1 and 2). The majority of these introduction events led to limited sequenced transmission (Fig. 2, Supplemental Fig. 1). Of these 100 events, 52 introductions were singletons, meaning that post-introduction transmission was sufficiently limited that we sequenced only one virus descended from the introduction event. Only 11 introduction events yielded a clade containing more than 10 sequenced infections, with Pango [17] lineage B.1.311 and B.1.243 strains accounting for the largest number of events (n=222 and 47, respectively) (Fig. 2). While not all SARS-CoV-2 positive specimens were sequenced, clades that transmit for longer periods of time within Humboldt County, or that contribute to larger outbreaks, are more likely to yield multiple sequenced isolates over time. These data suggest that the majority of introductions into Humboldt County were highly contained or self-limiting, while a small number (<10%) of introductions resulted in extended onward transmission within the county.

### Genomic surveillance validates and strengthens contact tracing during a farm-associated outbreak

In late-July 2020, multiple laboratory-confirmed cases of SARS-CoV-2 infections were identified in individuals reporting exposures to a commercial farm (Supplemental Fig. 3). Case-contact interviews revealed that two of the cases were roommates, and that there was extensive indoor contact with a team lead who was symptomatic between July 18 and July 20, 2020. The employee housing at the farm represented a high-contact congregate living facility, and 68 fellow employees were considered close contacts of cases and were ordered to quarantine. An additional 53 individuals who were not employees at the facility were also reported as close contacts and requested to quarantine. DHHS-PH encouraged exposed individuals who developed symptoms to seek testing at either the OptumServe location or at the local hospital to reduce the burden of travel. Between July 26, 2020 and October 29, 2020, 76 laboratory-confirmed cases were linked to the farm-associated outbreak; 61 cases occurred among employees of the facility and 15 cases occurred among members of the broader community.

**Fig. 3:**
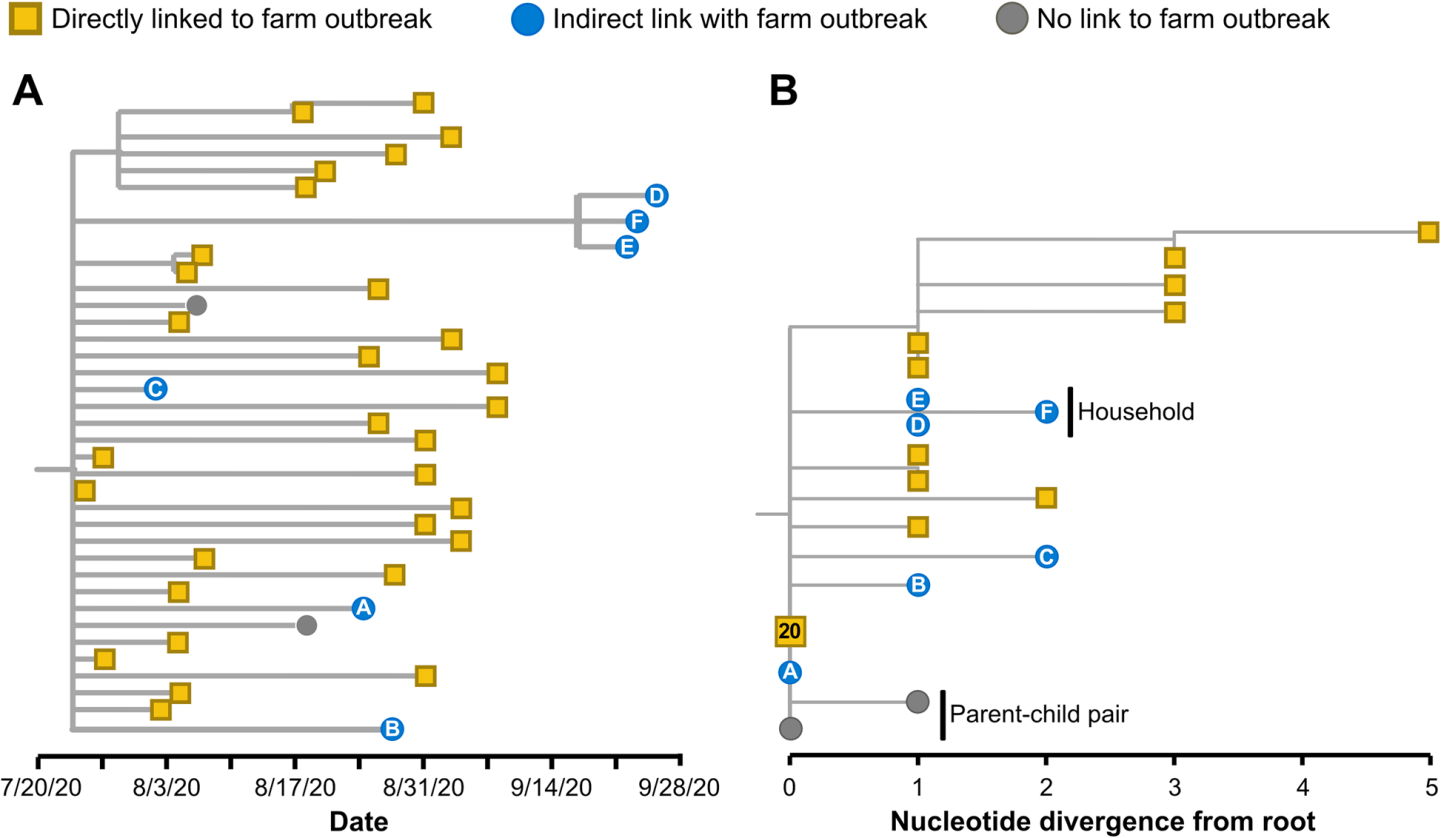
**A** Temporally-resolved phylogenetic tree showing the farm-associated outbreak clade. Viruses collected from individuals reporting an epidemiologic link to the farm are indicated as yellow squares, individuals with an indirect link to the farm are shown as blue circles, and individuals testing positive within the community with no reported connection with the farm are shown as grey circles. The indirect linkages are labeled A through F, and the nature of the link is described in text. **B** Maximum likelihood genetic divergence tree of the same clade as shown in panel **A**. Genome sequences that are identical are dispersed along the y-axis at the same location along the x-axis. Twenty identical sequences collected from employees at the farm are indicated as a single collapsed node on the tree

Genomic surveillance was initiated by DHHS-PH to characterize the outbreak, investigate potential epidemiological linkage between cases, and guide public health interventions. In total, 38 whole genome sequences were generated that grouped together within a clade defined by two substitutions (C18744T and G25699T). Thirty sequences were generated from diagnostic specimens collected from positive employees at the farm, while eight sequences were sampled from individuals reporting either an indirect link to the farm, or no contact with the farm whatsoever (Fig. 3).

The most basal genotype within this clade was detected in twenty specimens collected from employees of the farm, from one specimen from an individual with an indirect link to the farm, and from one individual reporting no connection to the farm at all (Fig. 3B). While viral genetic diversity accrued over the duration of the farm outbreak (top clade of genetic divergence tree in Fig. 3B), none of the unlinked or indirectly linked cases group within that clade.

Notably, this tree is consistent with two different epidemiologic scenarios. This pattern could have resulted from circulation of a SARS-CoV-2 lineage within the broader community that then moved into the farm, yielding a large outbreak that remained confined to the farm (directionality: broader community to farm). Alternatively, amplification of transmission during the farm outbreak could have contributed to spillover of transmission from the farm back into the broader Humboldt County community (directionality: farm to broader community).

We combined case interview data with the inferred tree to investigate these two plausible scenarios. Two cases, a parent and their child, reported no exposure to the farm or any employee of the farm (Fig. 3). Six cases occurred among individuals who did not work at the farm, but who had various degrees of connection with farm staff. Individual A (Fig. 3) lived with a farm employee. Individual B worked at a commercial dairy that reported some cases coinciding with the farm outbreak. Case interviews elucidated that a case at the dairy (not sequenced) lived with an employee of the farm (also not sequenced). Individuals D, E, and F shared a household. Individual F worked at the dairy; individuals D and E had no connection to the farm or the dairy beyond their contact with individual F. The combined genomic and contact-tracing data thereby suggest linkage between the farm outbreak and cases that occurred at the dairy, with further transmission into populations with no occupational exposure to either the farm or the dairy.

Whole genome sequencing helped support contact tracing efforts during this outbreak in various ways. Firstly, pathogen genomic data clearly linked infections among staff of the farm (Fig. 3), thereby validating information collected in case interviews. Secondly, genomic data indicated a link between the outbreak on the farm and cases detected at a local commercial dairy, a connection that had not initially been made from case interview data. Thirdly, for cases with multiple potential exposures, viral genomes enabled investigators to determine which exposure had likely resulted in transmission. Finally, investigators could use the genomic data to prospectively monitor for any new cases nesting within the farm outbreak genomic diversity, which allowed epidemiologists to more accurately determine when the outbreak had truly ended.

This outbreak was one of the first large, localized outbreaks that occurred in Humboldt County, and the experience shaped the public health response going forward. This outbreak demonstrated that rapid SARS-CoV-2 transmission could occur within congregate living facilities housing young and active individuals, and that such outbreaks could potentially seed transmission outside of the primary outbreak setting. In response, DHHS-PH implemented a specific testing protocol to monitor for and respond to outbreaks in congregate settings. Under this regime, individuals working or residing in congregate settings were tested for SARS-CoV-2 weekly as surveillance testing. If a positive test was confirmed, this triggered response testing. Contact tracing was initiated, and staff and residents were tested twice weekly (if possible) until a two week period of time with no additional positive tests had elapsed. Additionally, DHHS-PH devoted additional resources to working with congregate living facilities to reduce transmission risk, such as working with commercial agricultural settings to break apart housing into smaller pods and providing more rapid access to testing.

### Detection of de novo N501Y emergence within a skilled nursing facility outbreak

Like many parts of the United States, Humboldt County experienced a surge in COVID-19 cases during the late-fall and early-winter of 2020. Levels of community transmission were higher, increasing the probability of an introduction into congregate living settings at high risk for outbreaks. As part of standard testing procedures for congregate settings, on November 22, 2020 a SARS-CoV-2-positive specimen was collected from a staff member working in a skilled nursing facility, referred to here as Facility A. An outbreak was declared on November 23, 2020, which led to increased testing and contact tracing as per the congregate setting testing protocols described above. Over the course of the outbreak, all 72 residents of the facility tested positive for SARS-CoV-2 (100% attack rate), and 28 of the 98 staff members tested positive (28.6% attack rate). Thirteen residents of the facility died. No mortality was observed amongst infected staff. By January 16, 2021, no additional positive cases from Facility A had been recorded for two weeks, and the outbreak was declared over.

**Fig. 4:**
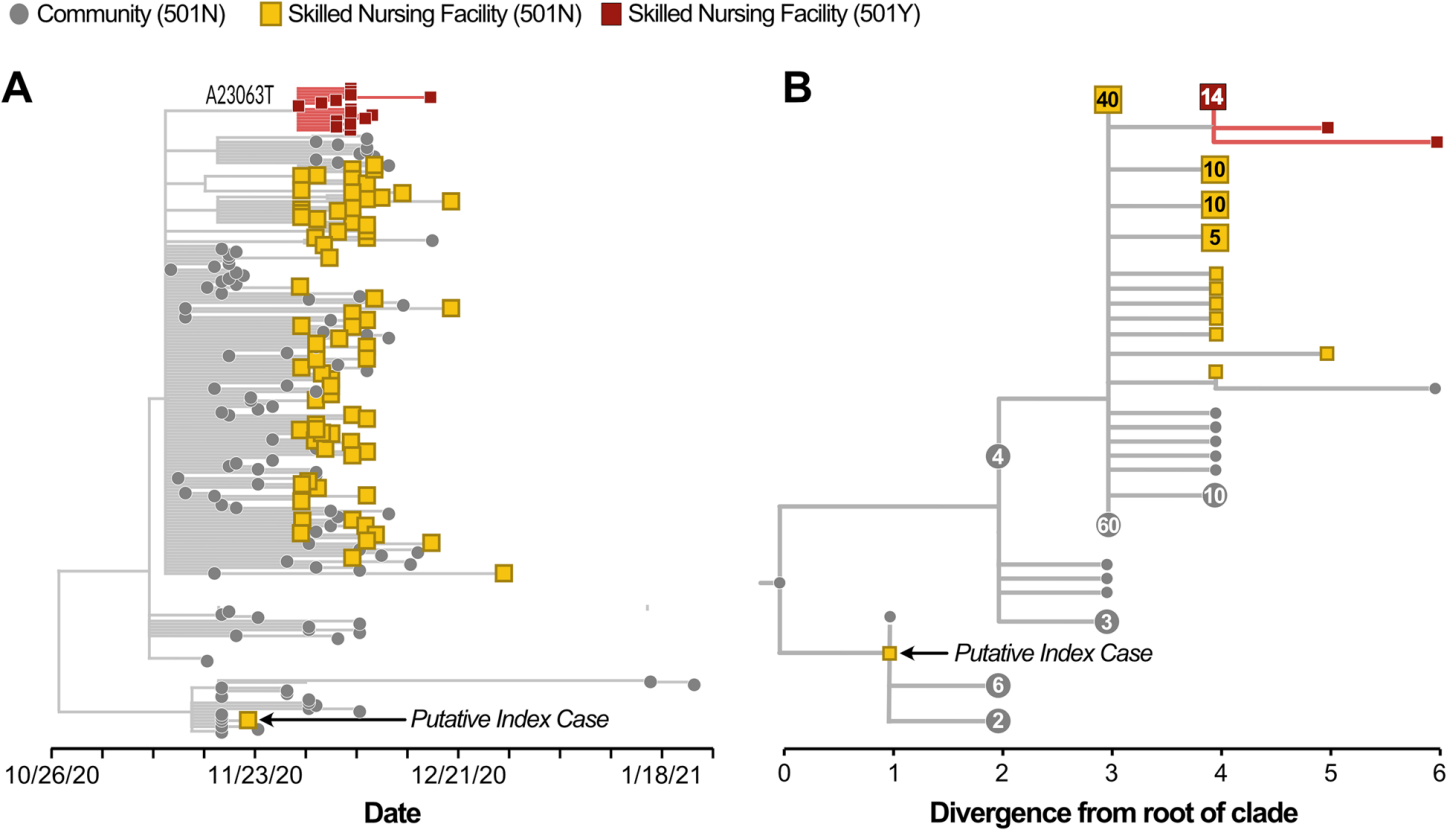
**A** Temporally-resolved phylogenetic tree showing cases sampled from either the SNF (residents and staff, squares) or the broader community (grey circles). The putative index case is annotated. While the community-associated lineage continued to circulate and was sampled in late-January 2021, the SNF-associated clade was not detected after the end of December 2020. **B** Genetic divergence tree indicating cases within the community (grey tips) and in the skilled nursing facility that had the wildtype N at site 501 in Spike (square yellow tips), and the emergent clade with a Y at site 501 (square maroon tips). The nucleotide substitution yielding the amino acid substitution is annotated on the tree. Square tips represent cases among either the staff or resident population at the SNF, while circular tips represent cases within the community. Large clades of identical genomes are collapsed, with either a square or circular tip, and are annotated with the number of identical genomes that the collapsed tip represents. The staff member that was the putative index case given contact tracing information is annotated

Routine genomic surveillance of SARS-CoV-2 infections occurring within Humboldt County throughout the fall provided crucial context for understanding this outbreak. In the lead-up to the outbreak in Facility A, a subset of community-associated SARS-CoV-2 cases had been sequenced as part of ongoing genomic surveillance (Fig. 4, grey tips). Furthermore, 89 out of the 100 positive specimens collected from staff and residents of Facility A were sequenced. The genome sequence from the postulated index case, the staff member who had tested positive on November 22, 2020, was more closely related to community cases of SARS-CoV-2 detected in late November 2020 than to sequences collected from other staff and residents at Facility A (Fig. 4B). Indeed, their viral genome sequence was diverged from the initial strain that circulated in Facility A by four nucleotide mutations, despite being sampled only one week before the earliest cases reported at Facility A. This finding suggests that this staff member did not seed the SNF outbreak. The genomic data thus supported an alternative mechanism of introduction into Facility A than was postulated given contact tracing data.

Genomic surveillance of the outbreak in Facility A also revealed the emergence and eradication of a lineage that had a *de novo* asparagine to tyrosine substitution in site 501 of the Spike protein (N501Y). This amino acid substitution is associated with increased binding affinity for the human ACE2 receptor [18], which could increase transmissibility. Notably, N501Y is present in three “variant of concern” lineages: B.1.1.7 [8], B.1.351 [19], and P.1 [20]. Of 89 samples sequenced during this outbreak, 16 samples shared this N501Y substitution (Fig. 4, indicated in maroon). The first sample containing this N501Y substitution was collected on November 29, 2020. Within this clade, 14 of the 16 sequences differed from the primary outbreak strain in Facility A by only the A23063T substitution that yielded the N501Y change. Two sequences showed additional diversity (Fig. 4B). One virus had an additional C to T substitution at site 19,273 which yielded a proline to serine substitution at site 1936 in ORF1b. A second sample showed two C to T mutations at sites 10,582 and 23,647.

Ongoing community genomic surveillance of positive cases verified eradication of this lineage from Humboldt County. Since December 29, 2020, 233 samples collected from SARS-CoV-2 cases in Humboldt County were sequenced, yet none of these samples grouped with, or were descended from, the 501 N or 501Y lineages that circulated in Facility A. Two cases sampled in January 2021 had viral genome sequences that were descended from the lineage that circulated in the community (Fig. 4 A). These data suggest that intervention efforts effectively eradicated Facility A’s outbreak lineages even as transmission within the wider community continued.

## Discussion

Here we have described the development of increased laboratory capacity and use of genomic surveillance to support the public health response to COVID-19 in a local health department. While genomic surveillance data have frequently been used to understand geographical patterns of infectious disease transmission, our work here demonstrates the utility of pathogen genomic sequence data for supporting actionable public health activities, such as contact tracing and outbreak response, within a rural county locale. Due to the rural nature of Humboldt County, there are generally longer turnaround times between sample collection and a test result because of the need to transport samples longer distances. Sample transport usually adds several days to the time necessary to receive a test result, and these delays can affect the ability to respond locally in a timely manner. To mitigate the impact of these issues, the HCPHL focused on modernizing their public health surveillance capabilities, including scaling up testing infrastructure and integrating viral genomic surveillance into response efforts.

These laboratory investments likely contributed significantly to DHHS-PH’s epidemiologic response efforts, thereby limiting community spread for many months. Combining traditional contact tracing with phylogenetic analysis of viral sequence data facilitated rapid identification and response in three important ways. First, sustained community transmission within the county could be actively monitored, instead of relying solely on state-wide or national data. Had DHHS-PH been reliant on state-wide or national data, several key opportunities to control local transmission may have been missed. Second, the ability to combine findings from case-contact interviews with genomic data allowed determination of which exposure event had resulted in transmission given multiple possible exposures, providing direct feedback on the efficacy of the overall response efforts. Finally, these new capabilities allowed early identification of introductions or emergence of variants of concern for immediate monitoring, containment, and local elimination.

The genomic surveillance findings in Humboldt County suggest that the majority of introductions into the county resulted in limited onward transmission and successful local control with only 11 (11%) of documented introduction events resulting in greater than 10 sequenced cases. Notably, this analysis is likely conservative due to the directionality of the bias in the phylogeographic reconstruction. If greater numbers of sequences from other geographic areas were added, they would likely interdigitate with, and potentially break apart, some of the clades inferred to circulate within Humboldt County. In this case, the number of discrete introductions into Humboldt County would be greater, and the amount of onward transmission observed post-introduction would decrease further. Thus, our estimate of at least 100 distinct introductions of SARS-CoV-2 to Humboldt County should be viewed as the minimal estimate of introduction frequency, and lineages may be even more self-limiting than observed in this study. Furthermore, we note that the tail of the clade size distribution may be skewed by testing policies. While outbreaks were not preferentially sequenced (HCPHL sent all SARS-CoV-2 positive specimens with Ct values less than 31 for sequencing), public health policies prioritized testing in congregate settings, likely increasing the numbers of tests conducted compared to testing in the broader community. Thus, even with the same sequencing criteria applied to all samples, outbreaks in congregate settings may have greater numbers of sequences because they were tested more densely, inflating the introduction clade size compared to more limited introductions.

Using this genomic surveillance system, we detected the emergence of a viral strain with a *de novo* N501Y substitution in the Spike protein. While the phenotypic characteristics of this substitution within this specific genetic context are not known, DHHS-PH’s response to this emergence event appears to have resulted in eradication of this lineage. This was feasible because a large proportion of positive diagnostic specimens collected from the surrounding community was consistently being sequenced. Monitoring these community samples in addition to specimens collected from the SNF where the substitution arose demonstrated that the lineage had been successfully contained to the facility and did not spill over and transmit within the broader community. To our knowledge, this is the first instance of a local health department within the United States detecting an emergent mutation of interest and documenting its eradication with genomic surveillance.

Genomic surveillance is still considered an advanced technique in public health and is often more accessible at higher levels of jurisdictional authority or in higher resource urban settings. This issue has less to do with implementation of laboratory protocols for conducting sequencing, which are increasingly accessible to most public health microbiology labs with molecular capabilities. Rather, this issue arises due to the unique challenges associated with genomic data management, analysis, and interpretation [21, 22]. However, our experience in Humboldt County shows the value in supporting genomic surveillance capacity at the local level and integrating it with event- and indicator-based surveillance efforts. Public health agencies at the local level are typically the frontline response to local public health issues. Thus, improving these agencies’ ability to generate, analyze and interpret genomic data rapidly makes the data more actionable for public health decision making. To support this objective, the California COVID Tracker Project is transitioning from providing genomic epidemiology as a service to supporting development of in-house genomic surveillance capacity for local public health departments. This effort includes hands-on training, education, and lowering analysis barriers by developing open-source software tools. Such partnerships can provide invaluable technology and knowledge transfer to local public health departments, which will foster representative, flexible, and responsive deployment of genomic epidemiology for surveillance and outbreak response based on local needs and priorities. If facilitated by a sustained increase in government funding, these partnership and capacity building models have the potential to facilitate genomic surveillance programs at the county level and modernize public health surveillance across all levels of jurisdictional authority.

## Additional files

**Additional file 1.**

**Additional file 2.**

## Data Availability

The *Augur* and *Auspice* components of the Nextstrain workflow are publicly available at https://github.com/nextstrain/. The SARS-CoV-2-specific Nextstrain phylogenetic pipeline is publicly available at https://github.com/nextstrain/ncov. The profile that specifies our particular parameters for this workflow is available at https://github.com/alliblk/ncov-humboldt. Scripts used in data analysis are also available at https://github.com/alliblk/ncov-humboldt. Whole genome sequences generated by CZ Biohub are available within GISAID and NCBI GenBank.
