# Supplementary material for: Using genomic epidemiology of SARS-CoV-2 to support contact tracing and public health surveillance in rural Humboldt County, California
Source: BMC Public Health. 2022 Mar 7;22:456. doi: 10.1186/s12889-022-12790-0 (PMC8900115; doi:10.1186/s12889-022-12790-0)
Supplement: Supplementary file 1 — Additional file 1. [file 12889_2022_12790_MOESM1_ESM.pdf]

Supplementary Information for "Using genomic epidemiology of SARS-CoV-2 to support contact tracing and public health surveillance in rural Humboldt County, California."

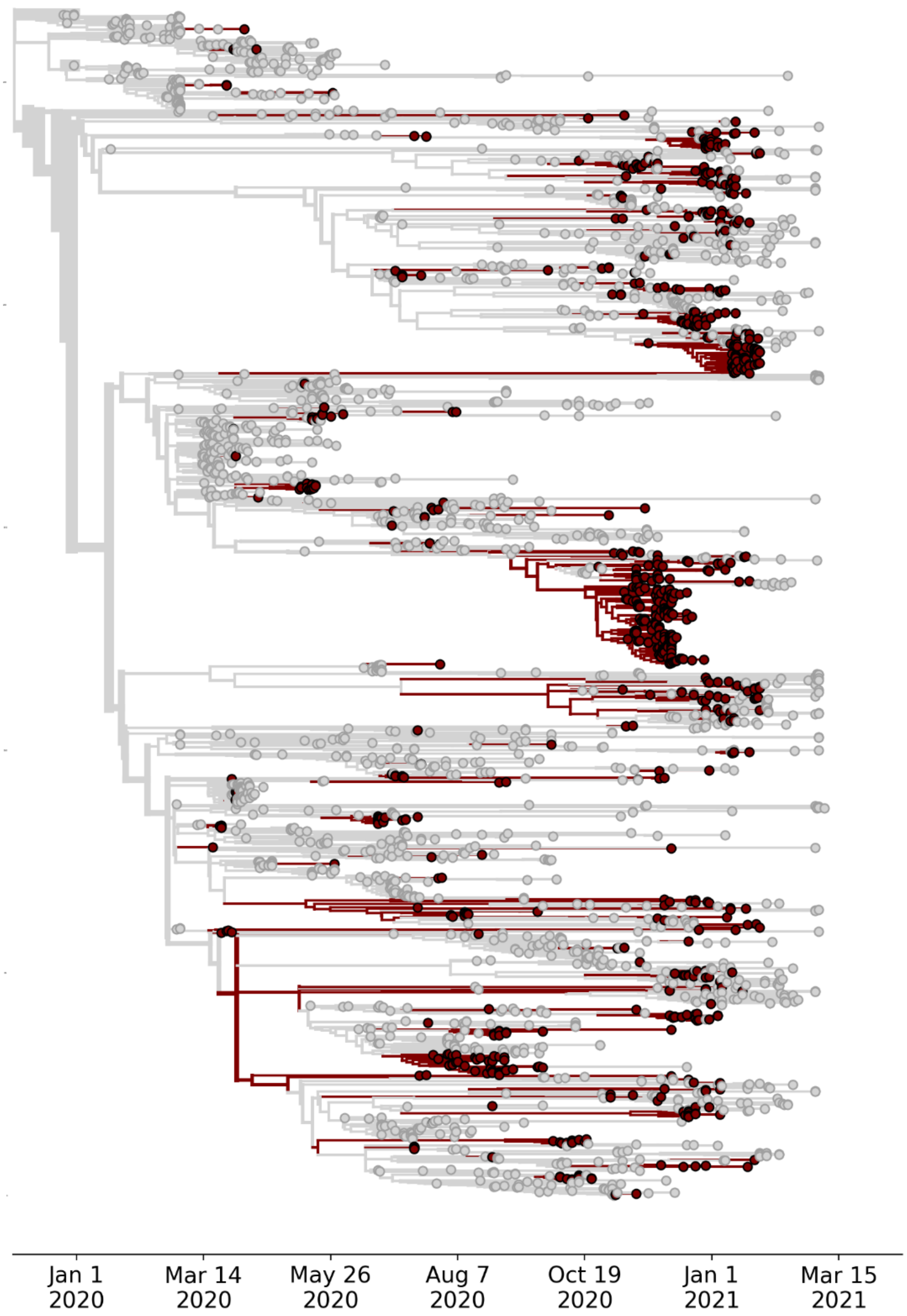

Supplemental Figure 1: Temporally-resolved phylogenetic tree of 2653 SARS-CoV-2 genomes, 853 of which were sampled from Humboldt County. Tips sampled from Humboldt County are indicated in maroon and tips sampled from other locations are colored in grey. Branches colored in maroon indicate inferred circulation within Humboldt County, and branches in grey indicate inferred transmission outside of Humboldt County.

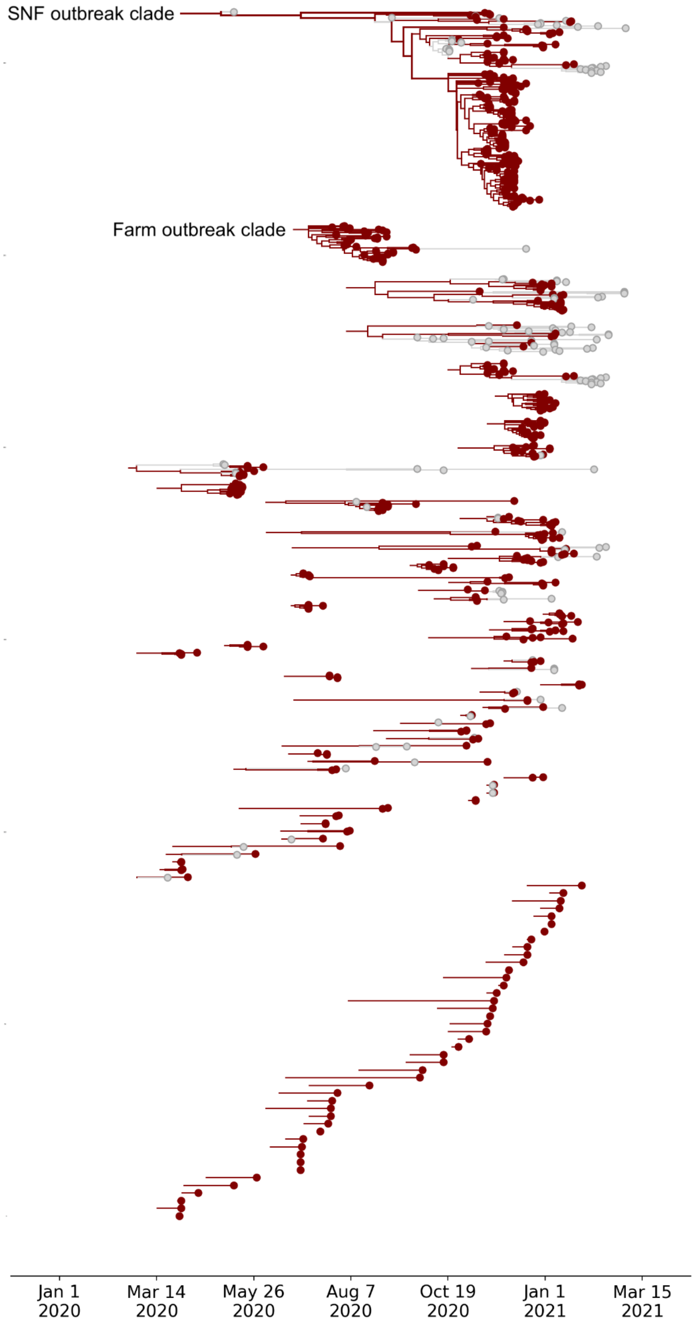

Supplemental Figure 2: Phylogenetic clades for 100 discrete lineages of SARS-CoV-2 introduced to Humboldt County. This visualization is derived from the phylogenetic tree in Supplemental Figure 1; in this figure we have simply taken away portions of the tree that circulated outside of Humboldt County. Here, each clade represents a unique introduction of SARS-CoV-2 to the county, and the length of the maroon branches indicate transmission duration within Humboldt County. Grey tips and branches represent viruses that descend from diversity that accrued while a clade was circulating in Humboldt County, but that were sampled outside of Humboldt County. Most introductions lead to limited transmission within Humboldt County, as evidenced by minimal genetic diversity of a clade sampled within the county. Clades associated with the farm outbreak and the SNF outbreak are annotated.

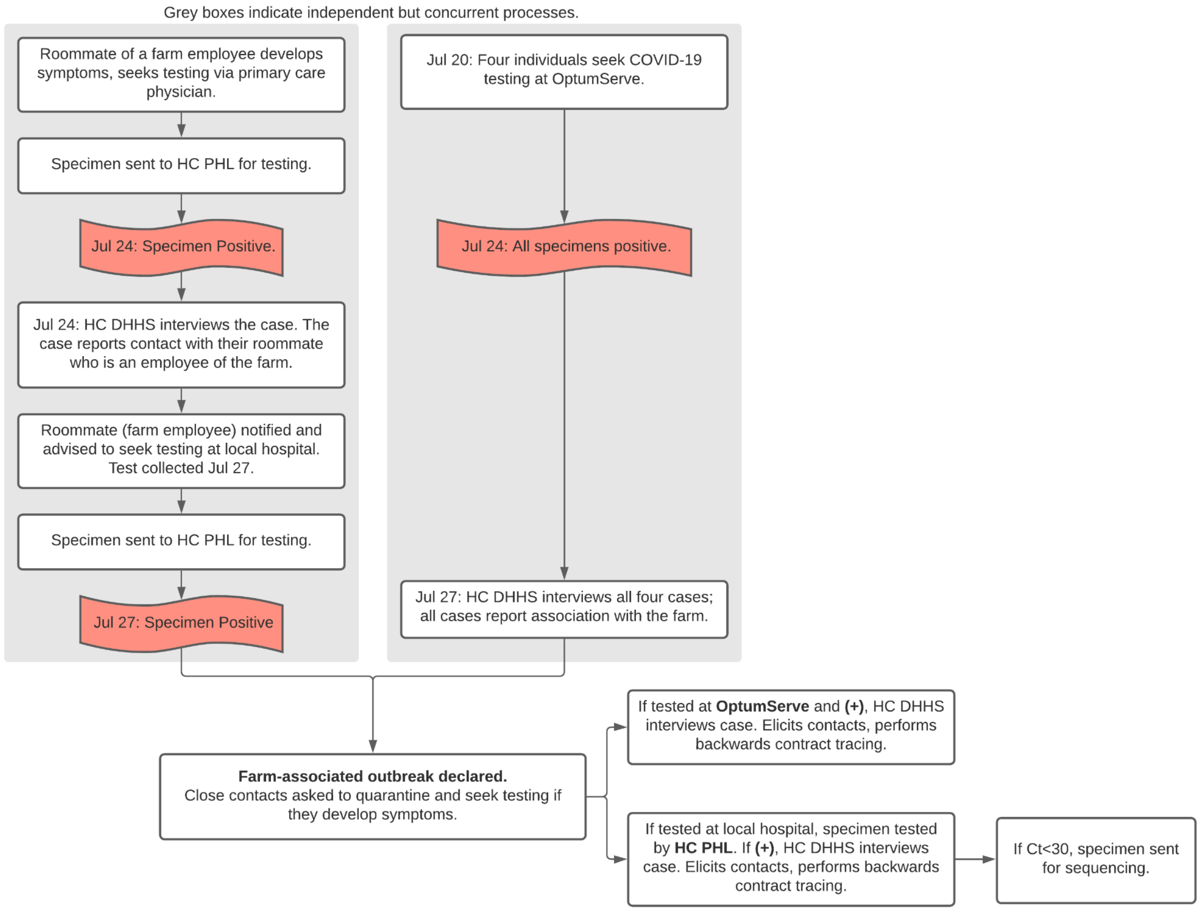

Supplemental Figure 3: Flowchart explaining how case interview data and sequence data were collected during the outbreak at a commercial farm in Humboldt County. The flowchart begins with the first confirmed positive case, and goes through actions taken and recommendations given by communicable disease investigators with the Humboldt County Department of Health and Human Services (HC DHHS) or by the Humboldt County Public Health Laboratory (HC PHL). The two grey boxes indicate independent processes that were co-occurring at the start of this outbreak.

Supplemental Table 1: Sequence data accession numbers. All sequences were submitted to GISAID as they were generated, and accession numbers are given. At the end of the study sequences were also deposited into NCBI GenBank. Some sequences accepted by GISAID were rejected by NCBI. In these cases, the GenBank accession number is given as “NA”.

**Sample_ID**	**GISAID_accession**	**GenBank_accession**
USA/CA-CZB-10785/2020	EPI_ISL_583046	MW276323
USA/CA-CZB-10786/2020	EPI_ISL_583047	MW276324
USA/CA-CZB-10788/2020	EPI_ISL_583048	MW276325
USA/CA-CZB-10789/2020	EPI_ISL_583049	MW276326
USA/CA-CZB-10790/2020	EPI_ISL_583050	MW276327
USA/CA-CZB-10791/2020	EPI_ISL_583051	MW276328
USA/CA-CZB-10792/2020	EPI_ISL_583052	MW276329
USA/CA-CZB-10793/2020	EPI_ISL_583053	MW276330
USA/CA-CZB-10794/2020	EPI_ISL_583054	MW276331
USA/CA-CZB-10795/2020	EPI_ISL_583055	MW276332
USA/CA-CZB-10796/2020	EPI_ISL_583056	MW276333
USA/CA-CZB-10797/2020	EPI_ISL_583057	MW276334
USA/CA-CZB-10798/2020	EPI_ISL_583058	MW276335
USA/CA-CZB-10799/2020	EPI_ISL_2659414	MZ842558
USA/CA-CZB-10800/2020	EPI_ISL_2659492	MZ842557
USA/CA-CZB-10801/2020	EPI_ISL_583059	MW276336
USA/CA-CZB-10802/2020	EPI_ISL_583060	MW276337
USA/CA-CZB-10803/2020	EPI_ISL_2659500	MZ842556
USA/CA-CZB-10804/2020	EPI_ISL_583061	MW276338
USA/CA-CZB-10805/2020	EPI_ISL_2659209	MZ842555
USA/CA-CZB-10808/2020	EPI_ISL_2659371	MZ842554
USA/CA-CZB-10809/2020	EPI_ISL_583062	MW276339
USA/CA-CZB-10810/2020	EPI_ISL_583063	MW276340
USA/CA-CZB-10811/2020	EPI_ISL_583064	MW276341
USA/CA-CZB-10812/2020	EPI_ISL_583065	MW276342

USA/CA-CZB-10813/2020	EPI_ISL_583066	MW276343
USA/CA-CZB-10815/2020	EPI_ISL_583067	MW276344
USA/CA-CZB-10817/2020	EPI_ISL_2659405	MZ842553
USA/CA-CZB-10818/2020	EPI_ISL_583068	MW276345
USA/CA-CZB-10819/2020	EPI_ISL_583069	MW276346
USA/CA-CZB-10820/2020	EPI_ISL_583070	MW276347
USA/CA-CZB-10821/2020	EPI_ISL_583071	MW276348
USA/CA-CZB-10822/2020	EPI_ISL_583072	MW276349
USA/CA-CZB-10823/2020	EPI_ISL_583073	MW276350
USA/CA-CZB-10824/2020	EPI_ISL_583074	MW276351
USA/CA-CZB-10825/2020	EPI_ISL_583075	MW276352
USA/CA-CZB-10826/2020	EPI_ISL_583076	MW276353
USA/CA-CZB-10827/2020	EPI_ISL_583077	MW276354
USA/CA-CZB-10828/2020	EPI_ISL_583078	MW276355
USA/CA-CZB-10829/2020	EPI_ISL_2758407	MZ842552
USA/CA-CZB-10830/2020	EPI_ISL_583079	MW276356
USA/CA-CZB-10831/2020	EPI_ISL_2659486	MZ842551
USA/CA-CZB-10832/2020	EPI_ISL_583080	MW276357
USA/CA-CZB-10833/2020	EPI_ISL_2659487	MZ842550
USA/CA-CZB-10834/2020	EPI_ISL_583081	MW276358
USA/CA-CZB-10835/2020	EPI_ISL_583082	MW276359
USA/CA-CZB-10836/2020	EPI_ISL_583083	MW276360
USA/CA-CZB-10837/2020	EPI_ISL_583084	MW276361
USA/CA-CZB-10838/2020	EPI_ISL_2758408	MZ842549
USA/CA-CZB-10839/2020	EPI_ISL_583085	MW276362
USA/CA-CZB-10841/2020	EPI_ISL_583086	MW276363
USA/CA-CZB-10842/2020	EPI_ISL_583087	MW276364
USA/CA-CZB-10843/2020	EPI_ISL_583088	MW276365
USA/CA-CZB-10844/2020	EPI_ISL_583089	MW276366

USA/CA-CZB-10845/2020	EPI_ISL_583090	MZ842548
USA/CA-CZB-10846/2020	EPI_ISL_583091	MW276367
USA/CA-CZB-10847/2020	EPI_ISL_583092	MW276368
USA/CA-CZB-10848/2020	EPI_ISL_583093	MW276369
USA/CA-CZB-10849/2020	EPI_ISL_583094	MW276370
USA/CA-CZB-10850/2020	EPI_ISL_583095	MW276371
USA/CA-CZB-10851/2020	EPI_ISL_583096	MW276372
USA/CA-CZB-10852/2020	EPI_ISL_583097	MW276373
USA/CA-CZB-10854/2020	EPI_ISL_583098	MW276374
USA/CA-CZB-10855/2020	EPI_ISL_583099	MW276375
USA/CA-CZB-10856/2020	EPI_ISL_583100	MW276376
USA/CA-CZB-10858/2020	EPI_ISL_583101	MZ842547
USA/CA-CZB-10860/2020	EPI_ISL_583102	MW276377
USA/CA-CZB-10861/2020	EPI_ISL_583103	MW276378
USA/CA-CZB-10862/2020	EPI_ISL_583104	MW276379
USA/CA-CZB-10863/2020	EPI_ISL_583105	MW276380
USA/CA-CZB-10864/2020	EPI_ISL_2758409	NA
USA/CA-CZB-10865/2020	EPI_ISL_583106	MW276381
USA/CA-CZB-10866/2020	EPI_ISL_583107	MW276382
USA/CA-CZB-10867/2020	EPI_ISL_583108	MW276383
USA/CA-CZB-10868/2020	EPI_ISL_583109	MW276384
USA/CA-CZB-10870/2020	EPI_ISL_583110	MW276385
USA/CA-CZB-10871/2020	EPI_ISL_583111	MW276386
USA/CA-CZB-10872/2020	EPI_ISL_583112	MW276387
USA/CA-CZB-10873/2020	EPI_ISL_583113	MW276388
USA/CA-CZB-10874/2020	EPI_ISL_583114	MW276389
USA/CA-CZB-10876/2020	EPI_ISL_583115	MW276390
USA/CA-CZB-10878/2020	EPI_ISL_583116	MW276391
USA/CA-CZB-10879/2020	EPI_ISL_583117	MW276392

USA/CA-CZB-10880/2020	EPI_ISL_583118	MW276393
USA/CA-CZB-10881/2020	EPI_ISL_583119	MW276394
USA/CA-CZB-10885/2020	EPI_ISL_583120	MW276395
USA/CA-CZB-10887/2020	EPI_ISL_583121	MW276396
USA/CA-CZB-10889/2020	EPI_ISL_583122	MW276397
USA/CA-CZB-10891/2020	EPI_ISL_583123	MW276398
USA/CA-CZB-10892/2020	EPI_ISL_583124	MW276399
USA/CA-CZB-10893/2020	EPI_ISL_2658689	NA
USA/CA-CZB-10895/2020	EPI_ISL_583125	MW276400
USA/CA-CZB-10896/2020	EPI_ISL_583126	MW276401
USA/CA-CZB-10897/2020	EPI_ISL_583127	MW276402
USA/CA-CZB-10898/2020	EPI_ISL_583128	MW276403
USA/CA-CZB-10899/2020	EPI_ISL_583129	MW276404
USA/CA-CZB-10901/2020	EPI_ISL_583130	MW276405
USA/CA-CZB-10902/2020	EPI_ISL_583131	MW276406
USA/CA-CZB-10903/2020	EPI_ISL_583132	MW276407
USA/CA-CZB-10904/2020	EPI_ISL_583133	MW276408
USA/CA-CZB-10905/2020	EPI_ISL_583134	MW276409
USA/CA-CZB-10906/2020	EPI_ISL_583135	MW276410
USA/CA-CZB-10907/2020	EPI_ISL_583136	MW276411
USA/CA-CZB-10908/2020	EPI_ISL_583137	MW276412
USA/CA-CZB-10909/2020	EPI_ISL_583138	MW276413
USA/CA-CZB-10910/2020	EPI_ISL_583139	MW276414
USA/CA-CZB-10911/2020	EPI_ISL_583140	MW276415
USA/CA-CZB-10912/2020	EPI_ISL_583141	MW276416
USA/CA-CZB-10913/2020	EPI_ISL_583142	MW276417
USA/CA-CZB-10914/2020	EPI_ISL_583143	MW276418
USA/CA-CZB-10915/2020	EPI_ISL_583144	MW276419
USA/CA-CZB-10916/2020	EPI_ISL_583145	MW276420

USA/CA-CZB-10917/2020	EPI_ISL_583146	MW276421
USA/CA-CZB-10918/2020	EPI_ISL_583147	MW276422
USA/CA-CZB-10919/2020	EPI_ISL_583148	MW276423
USA/CA-CZB-10920/2020	EPI_ISL_583149	MW276424
USA/CA-CZB-10921/2020	EPI_ISL_583150	MW276425
USA/CA-CZB-10922/2020	EPI_ISL_583151	MW276426
USA/CA-CZB-10923/2020	EPI_ISL_583152	MW276427
USA/CA-CZB-10924/2020	EPI_ISL_583153	MW276428
USA/CA-CZB-10925/2020	EPI_ISL_583154	MW276429
USA/CA-CZB-10926/2020	EPI_ISL_583155	MW276430
USA/CA-CZB-10927/2020	EPI_ISL_583156	MW276431
USA/CA-CZB-10928/2020	EPI_ISL_583157	MW276432
USA/CA-CZB-10929/2020	EPI_ISL_583158	MW276433
USA/CA-CZB-10930/2020	EPI_ISL_583159	MW276434
USA/CA-CZB-10931/2020	EPI_ISL_583160	MW276435
USA/CA-CZB-10932/2020	EPI_ISL_583161	MW276436
USA/CA-CZB-10933/2020	EPI_ISL_583162	MW276437
USA/CA-CZB-10934/2020	EPI_ISL_583163	MW276438
USA/CA-CZB-10935/2020	EPI_ISL_583164	MW276439
USA/CA-CZB-10936/2020	EPI_ISL_583165	MW276440
USA/CA-CZB-10937/2020	EPI_ISL_583166	MW276441
USA/CA-CZB-10938/2020	EPI_ISL_583167	MW276442
USA/CA-CZB-10939/2020	EPI_ISL_583168	MW276443
USA/CA-CZB-10940/2020	EPI_ISL_583169	MW276444
USA/CA-CZB-10941/2020	EPI_ISL_583170	MW276445
USA/CA-CZB-1278/2020	EPI_ISL_454636	MT533233
USA/CA-CZB-1279/2020	EPI_ISL_454637	MT533234
USA/CA-CZB-1280/2020	EPI_ISL_454638	MT533235
USA/CA-CZB-1281/2020	EPI_ISL_454639	MT533236

USA/CA-CZB-1282/2020	EPI_ISL_454640	MT533237
USA/CA-CZB-1283/2020	EPI_ISL_454641	MT533238
USA/CA-CZB-14715/2020	EPI_ISL_738785	NA
USA/CA-CZB-14716/2020	EPI_ISL_738778	MW565247
USA/CA-CZB-14717/2020	EPI_ISL_739011	MW565246
USA/CA-CZB-14718/2020	EPI_ISL_2658704	NA
USA/CA-CZB-14721/2020	EPI_ISL_739489	MW565245
USA/CA-CZB-14722/2020	EPI_ISL_739264	NA
USA/CA-CZB-14724/2020	EPI_ISL_739086	MW565244
USA/CA-CZB-14725/2020	EPI_ISL_2659408	MZ842401
USA/CA-CZB-14727/2020	EPI_ISL_739297	MW565243
USA/CA-CZB-14728/2020	EPI_ISL_2659346	MZ842400
USA/CA-CZB-14729/2020	EPI_ISL_739571	MW565242
USA/CA-CZB-14730/2020	EPI_ISL_739366	MW565241
USA/CA-CZB-14731/2020	EPI_ISL_2659403	MZ842399
USA/CA-CZB-14733/2020	EPI_ISL_2659591	MZ842398
USA/CA-CZB-14734/2020	EPI_ISL_738988	MW565240
USA/CA-CZB-14735/2020	EPI_ISL_2659461	MZ842397
USA/CA-CZB-14736/2020	EPI_ISL_739311	MW565239
USA/CA-CZB-14737/2020	EPI_ISL_739533	MW565238
USA/CA-CZB-14739/2020	EPI_ISL_2659260	MZ842396
USA/CA-CZB-14740/2020	EPI_ISL_2659385	MZ842395
USA/CA-CZB-14741/2020	EPI_ISL_739177	MW565237
USA/CA-CZB-14742/2020	EPI_ISL_738961	MW565236
USA/CA-CZB-14743/2020	EPI_ISL_739558	MW565235
USA/CA-CZB-14744/2020	EPI_ISL_2659205	MZ842394
USA/CA-CZB-14745/2020	EPI_ISL_738530	MW565234
USA/CA-CZB-14746/2020	EPI_ISL_2659549	MZ842393
USA/CA-CZB-14747/2020	EPI_ISL_2659638	MZ842392

USA/CA-CZB-14748/2020	EPI_ISL_738879	MW565233
USA/CA-CZB-14749/2020	EPI_ISL_738756	MW565232
USA/CA-CZB-14750/2020	EPI_ISL_739225	MW565231
USA/CA-CZB-14751/2020	EPI_ISL_738951	MW565230
USA/CA-CZB-14752/2020	EPI_ISL_739148	MW565229
USA/CA-CZB-14754/2020	EPI_ISL_738947	MW565227
USA/CA-CZB-14755/2020	EPI_ISL_739433	MW565226
USA/CA-CZB-14756/2020	EPI_ISL_739530	MW565225
USA/CA-CZB-14757/2020	EPI_ISL_739442	MZ842391
USA/CA-CZB-14758/2020	EPI_ISL_738953	MZ842390
USA/CA-CZB-14759/2020	EPI_ISL_738832	MW565224
USA/CA-CZB-14760/2020	EPI_ISL_739052	MW565223
USA/CA-CZB-14767/2020	EPI_ISL_739051	MW565217
USA/CA-CZB-14768/2020	EPI_ISL_739272	MW565216
USA/CA-CZB-14769/2020	EPI_ISL_738844	MW565215
USA/CA-CZB-14770/2020	EPI_ISL_738742	MW565214
USA/CA-CZB-14771/2020	EPI_ISL_738852	MW565213
USA/CA-CZB-14772/2020	EPI_ISL_739411	MW565212
USA/CA-CZB-14773/2020	EPI_ISL_738827	MW565211
USA/CA-CZB-14774/2020	EPI_ISL_738600	MW565210
USA/CA-CZB-14775/2020	EPI_ISL_739527	MW565209
USA/CA-CZB-14777/2020	EPI_ISL_739330	MW565208
USA/CA-CZB-14778/2020	EPI_ISL_738912	MW565207
USA/CA-CZB-14779/2020	EPI_ISL_738795	MW565206
USA/CA-CZB-14780/2020	EPI_ISL_739367	MW565205
USA/CA-CZB-14781/2020	EPI_ISL_738759	MW565204
USA/CA-CZB-14782/2020	EPI_ISL_738985	MW565203
USA/CA-CZB-14783/2020	EPI_ISL_738779	MW565202
USA/CA-CZB-14784/2020	EPI_ISL_738810	MW565201

USA/CA-CZB-14785/2020	EPI_ISL_738913	MW565200
USA/CA-CZB-14786/2020	EPI_ISL_739553	MW565199
USA/CA-CZB-14788/2020	EPI_ISL_739194	MW565198
USA/CA-CZB-14789/2020	EPI_ISL_739621	MW565197
USA/CA-CZB-14790/2020	EPI_ISL_739525	MW565196
USA/CA-CZB-14791/2020	EPI_ISL_738923	MW565195
USA/CA-CZB-14792/2020	EPI_ISL_739032	MW565194
USA/CA-CZB-14793/2020	EPI_ISL_739447	MW565193
USA/CA-CZB-14795/2020	EPI_ISL_739318	MW565192
USA/CA-CZB-14796/2020	EPI_ISL_739542	MW565191
USA/CA-CZB-14797/2020	EPI_ISL_739389	MW565190
USA/CA-CZB-14798/2020	EPI_ISL_2658662	NA
USA/CA-CZB-14799/2020	EPI_ISL_739210	MW565189
USA/CA-CZB-14801/2020	EPI_ISL_739043	MW565188
USA/CA-CZB-14802/2020	EPI_ISL_738806	MW565187
USA/CA-CZB-14803/2020	EPI_ISL_739112	MW565186
USA/CA-CZB-14804/2020	EPI_ISL_739611	MW565185
USA/CA-CZB-14805/2020	EPI_ISL_738849	MW565184
USA/CA-CZB-14806/2020	EPI_ISL_739436	MW565183
USA/CA-CZB-14807/2020	EPI_ISL_738902	MZ842389
USA/CA-CZB-14808/2020	EPI_ISL_739287	MW565182
USA/CA-CZB-14809/2020	EPI_ISL_739313	MW565181
USA/CA-CZB-14810/2020	EPI_ISL_739240	MW565180
USA/CA-CZB-14812/2020	EPI_ISL_739000	MW565179
USA/CA-CZB-14813/2020	EPI_ISL_739228	MW565178
USA/CA-CZB-14814/2020	EPI_ISL_739531	MW565177
USA/CA-CZB-14815/2020	EPI_ISL_738917	MW565176
USA/CA-CZB-14816/2020	EPI_ISL_739170	MW565175
USA/CA-CZB-14817/2020	EPI_ISL_739001	MW565174

USA/CA-CZB-14818/2020	EPI_ISL_739114	MW565173
USA/CA-CZB-14819/2020	EPI_ISL_739007	MW565172
USA/CA-CZB-14820/2020	EPI_ISL_739242	MW565171
USA/CA-CZB-14821/2020	EPI_ISL_739651	MW565170
USA/CA-CZB-14822/2020	EPI_ISL_738504	MW565169
USA/CA-CZB-14823/2020	EPI_ISL_738875	MW565168
USA/CA-CZB-14824/2020	EPI_ISL_738980	MW565167
USA/CA-CZB-14825/2020	EPI_ISL_738865	MW565166
USA/CA-CZB-14826/2020	EPI_ISL_739405	MW565165
USA/CA-CZB-14827/2020	EPI_ISL_739403	MW565164
USA/CA-CZB-14828/2020	EPI_ISL_738938	MW565163
USA/CA-CZB-14829/2020	EPI_ISL_738807	MW565162
USA/CA-CZB-14830/2020	EPI_ISL_739475	MW565161
USA/CA-CZB-14831/2020	EPI_ISL_739417	MW565160
USA/CA-CZB-14832/2020	EPI_ISL_739123	MW565159
USA/CA-CZB-14833/2020	EPI_ISL_738801	MW565158
USA/CA-CZB-14834/2020	EPI_ISL_739274	MW565157
USA/CA-CZB-14835/2020	EPI_ISL_738563	MW565156
USA/CA-CZB-14836/2020	EPI_ISL_738969	MZ842388
USA/CA-CZB-14837/2020	EPI_ISL_739461	MW565155
USA/CA-CZB-14838/2020	EPI_ISL_739425	MW565154
USA/CA-CZB-14839/2020	EPI_ISL_738651	MW565153
USA/CA-CZB-14840/2020	EPI_ISL_738539	MW565152
USA/CA-CZB-14841/2020	EPI_ISL_739299	MW565151
USA/CA-CZB-14842/2020	EPI_ISL_739183	MW565150
USA/CA-CZB-14844/2020	EPI_ISL_739585	MW565149
USA/CA-CZB-14845/2020	EPI_ISL_738649	MW565148
USA/CA-CZB-14846/2020	EPI_ISL_739106	MW565147
USA/CA-CZB-14847/2020	EPI_ISL_738929	MW565146

USA/CA-CZB-14848/2020	EPI_ISL_738647	MW565145
USA/CA-CZB-14849/2020	EPI_ISL_738999	MW565144
USA/CA-CZB-14850/2020	EPI_ISL_738854	MZ842387
USA/CA-CZB-14851/2020	EPI_ISL_739452	MW565143
USA/CA-CZB-14852/2020	EPI_ISL_739418	MW565142
USA/CA-CZB-14853/2020	EPI_ISL_739155	MW565141
USA/CA-CZB-14854/2020	EPI_ISL_739126	MW565140
USA/CA-CZB-14855/2020	EPI_ISL_738958	MW565139
USA/CA-CZB-14856/2020	EPI_ISL_739499	MW565138
USA/CA-CZB-14857/2020	EPI_ISL_738809	MW565137
USA/CA-CZB-14858/2020	EPI_ISL_738505	MW565136
USA/CA-CZB-14859/2020	EPI_ISL_739250	MW565135
USA/CA-CZB-14860/2020	EPI_ISL_739608	MZ842386
USA/CA-CZB-14861/2020	EPI_ISL_739159	MW565134
USA/CA-CZB-14862/2020	EPI_ISL_739198	MW565133
USA/CA-CZB-14864/2020	EPI_ISL_739235	MW565132
USA/CA-CZB-14865/2020	EPI_ISL_739131	MW565131
USA/CA-CZB-14866/2020	EPI_ISL_739269	MW565130
USA/CA-CZB-1488/2020	EPI_ISL_468438	MT628172
USA/CA-CZB-1489/2020	EPI_ISL_468439	NA
USA/CA-CZB-1490/2020	EPI_ISL_468440	MT628173
USA/CA-CZB-1491/2020	EPI_ISL_468441	NA
USA/CA-CZB-1492/2020	EPI_ISL_468442	NA
USA/CA-CZB-1493/2020	EPI_ISL_468443	NA
USA/CA-CZB-1494/2020	EPI_ISL_468444	NA
USA/CA-CZB-1495/2020	EPI_ISL_468445	NA
USA/CA-CZB-1497/2020	EPI_ISL_468446	MT628174
USA/CA-CZB-1498/2020	EPI_ISL_468447	MT628175
USA/CA-CZB-1499/2020	EPI_ISL_468448	MT628176

USA/CA-CZB-1502/2020	EPI_ISL_468449	MT628177
USA/CA-CZB-1503/2020	EPI_ISL_468450	MT628178
USA/CA-CZB-1504/2020	EPI_ISL_468451	MT628179
USA/CA-CZB-1505/2020	EPI_ISL_468452	MT628180
USA/CA-CZB-1506/2020	EPI_ISL_468453	MT628181
USA/CA-CZB-1507/2020	EPI_ISL_468454	MT628182
USA/CA-CZB-1508/2020	EPI_ISL_468455	MT628183
USA/CA-CZB-1509/2020	EPI_ISL_468456	MT628184
USA/CA-CZB-1510/2020	EPI_ISL_468457	MT628185
USA/CA-CZB-1511/2020	EPI_ISL_468458	MT628186
USA/CA-CZB-1512/2020	EPI_ISL_468459	MT628187
USA/CA-CZB-1513/2020	EPI_ISL_468460	MT628188
USA/CA-CZB-1514/2020	EPI_ISL_468461	MT628189
USA/CA-CZB-17317/2020	EPI_ISL_955496	MW702500
USA/CA-CZB-17319/2020	EPI_ISL_955497	MW702499
USA/CA-CZB-17320/2020	EPI_ISL_1027535	MW703127
USA/CA-CZB-17321/2020	EPI_ISL_955498	MW702498
USA/CA-CZB-17322/2020	EPI_ISL_955499	MW702497
USA/CA-CZB-17323/2020	EPI_ISL_955500	MW702496
USA/CA-CZB-17324/2020	EPI_ISL_955501	MW702495
USA/CA-CZB-17325/2020	EPI_ISL_955502	MW702494
USA/CA-CZB-17326/2020	EPI_ISL_955503	MW702493
USA/CA-CZB-17327/2020	EPI_ISL_955504	MW702492
USA/CA-CZB-17329/2020	EPI_ISL_1027536	MW703126
USA/CA-CZB-17331/2020	EPI_ISL_955505	MW702491
USA/CA-CZB-17332/2020	EPI_ISL_955506	MW702490
USA/CA-CZB-17333/2020	EPI_ISL_955507	MW702489
USA/CA-CZB-17334/2020	EPI_ISL_955508	MW702488
USA/CA-CZB-17335/2020	EPI_ISL_955509	MW702487

USA/CA-CZB-17336/2020	EPI_ISL_955510	MW702486
USA/CA-CZB-17337/2020	EPI_ISL_955511	MW702485
USA/CA-CZB-17338/2020	EPI_ISL_955512	MW702484
USA/CA-CZB-17339/2020	EPI_ISL_955513	MW702483
USA/CA-CZB-17340/2020	EPI_ISL_955514	MW702482
USA/CA-CZB-17341/2020	EPI_ISL_1027537	MW703125
USA/CA-CZB-17342/2020	EPI_ISL_955515	MW702481
USA/CA-CZB-17343/2020	EPI_ISL_955516	MW702480
USA/CA-CZB-17344/2020	EPI_ISL_955517	MW702479
USA/CA-CZB-17345/2020	EPI_ISL_955518	MW702478
USA/CA-CZB-17346/2020	EPI_ISL_955519	MW702477
USA/CA-CZB-17347/2020	EPI_ISL_955520	MW702476
USA/CA-CZB-17348/2020	EPI_ISL_1027538	MW703124
USA/CA-CZB-17349/2020	EPI_ISL_955521	MW702475
USA/CA-CZB-17350/2020	EPI_ISL_955522	MW702474
USA/CA-CZB-17351/2020	EPI_ISL_955523	MW702473
USA/CA-CZB-17352/2020	EPI_ISL_955524	MW702472
USA/CA-CZB-17353/2020	EPI_ISL_955525	MW702471
USA/CA-CZB-17354/2020	EPI_ISL_2658668	NA
USA/CA-CZB-17355/2020	EPI_ISL_955526	MW702470
USA/CA-CZB-17356/2020	EPI_ISL_955527	MW702469
USA/CA-CZB-17357/2020	EPI_ISL_955528	MW702468
USA/CA-CZB-17358/2020	EPI_ISL_955529	MW702467
USA/CA-CZB-17359/2020	EPI_ISL_955530	MW702466
USA/CA-CZB-17360/2020	EPI_ISL_955531	MW702465
USA/CA-CZB-17361/2020	EPI_ISL_955532	MW702464
USA/CA-CZB-17362/2020	EPI_ISL_955533	MW702463
USA/CA-CZB-17363/2020	EPI_ISL_955534	MW702462
USA/CA-CZB-17364/2020	EPI_ISL_955535	MW702461

USA/CA-CZB-17365/2020	EPI_ISL_955536	MW702460
USA/CA-CZB-17366/2020	EPI_ISL_955537	MW702459
USA/CA-CZB-17367/2020	EPI_ISL_955538	MW702458
USA/CA-CZB-17368/2020	EPI_ISL_955539	MW702457
USA/CA-CZB-17369/2020	EPI_ISL_955540	MW702456
USA/CA-CZB-17370/2020	EPI_ISL_955541	MW702455
USA/CA-CZB-17371/2020	EPI_ISL_2659259	MW702454
USA/CA-CZB-17372/2020	EPI_ISL_955542	MW702453
USA/CA-CZB-17373/2020	EPI_ISL_2659308	MW702452
USA/CA-CZB-17374/2020	EPI_ISL_955543	MW702451
USA/CA-CZB-17375/2020	EPI_ISL_955544	MW702450
USA/CA-CZB-17376/2020	EPI_ISL_955545	MW702449
USA/CA-CZB-17377/2020	EPI_ISL_955546	MW702448
USA/CA-CZB-17378/2020	EPI_ISL_955547	MW702447
USA/CA-CZB-17379/2020	EPI_ISL_955548	MW702446
USA/CA-CZB-17380/2020	EPI_ISL_1027540	MW703123
USA/CA-CZB-17381/2020	EPI_ISL_955549	MW702445
USA/CA-CZB-17382/2020	EPI_ISL_955550	MW702444
USA/CA-CZB-17383/2020	EPI_ISL_955551	MW702443
USA/CA-CZB-17384/2020	EPI_ISL_955552	MW702442
USA/CA-CZB-17385/2020	EPI_ISL_955553	MW702441
USA/CA-CZB-17386/2020	EPI_ISL_955554	MW702440
USA/CA-CZB-17387/2020	EPI_ISL_955555	MW702439
USA/CA-CZB-17388/2020	EPI_ISL_1027539	MW703122
USA/CA-CZB-17389/2020	EPI_ISL_955556	MW702438
USA/CA-CZB-17390/2020	EPI_ISL_955557	MW702437
USA/CA-CZB-17393/2020	EPI_ISL_1027541	MW703121
USA/CA-CZB-17394/2020	EPI_ISL_955558	NA
USA/CA-CZB-17395/2020	EPI_ISL_955559	MW702436

USA/CA-CZB-17397/2020	EPI_ISL_955560	MW702435
USA/CA-CZB-19111/2020	EPI_ISL_979710	MZ842239
USA/CA-CZB-19112/2020	EPI_ISL_979711	MW701299
USA/CA-CZB-19113/2020	EPI_ISL_979712	MW701298
USA/CA-CZB-19114/2020	EPI_ISL_979713	MW701297
USA/CA-CZB-19115/2020	EPI_ISL_979714	MW701296
USA/CA-CZB-19116/2020	EPI_ISL_979715	MW701295
USA/CA-CZB-19117/2020	EPI_ISL_979716	MW701294
USA/CA-CZB-19118/2020	EPI_ISL_979717	MW701293
USA/CA-CZB-19119/2020	EPI_ISL_979718	MW701292
USA/CA-CZB-19120/2020	EPI_ISL_2659251	MW701291
USA/CA-CZB-19121/2020	EPI_ISL_979719	MW701290
USA/CA-CZB-19123/2020	EPI_ISL_2659384	MW701289
USA/CA-CZB-19124/2020	EPI_ISL_979720	MW701288
USA/CA-CZB-19125/2020	EPI_ISL_979721	MW701287
USA/CA-CZB-19126/2020	EPI_ISL_979722	MW701286
USA/CA-CZB-19127/2020	EPI_ISL_979723	MW701285
USA/CA-CZB-19128/2020	EPI_ISL_979724	MW701284
USA/CA-CZB-19129/2020	EPI_ISL_979725	MW701283
USA/CA-CZB-19132/2020	EPI_ISL_979726	MW701282
USA/CA-CZB-19133/2020	EPI_ISL_979727	MW701281
USA/CA-CZB-19134/2020	EPI_ISL_979728	MW701280
USA/CA-CZB-19135/2020	EPI_ISL_979729	MW701279
USA/CA-CZB-19136/2020	EPI_ISL_979730	MW701278
USA/CA-CZB-19137/2020	EPI_ISL_979731	MW701277
USA/CA-CZB-19138/2020	EPI_ISL_979732	MW701276
USA/CA-CZB-19139/2020	EPI_ISL_979733	MW701275
USA/CA-CZB-19140/2020	EPI_ISL_979734	MW701274
USA/CA-CZB-19141/2020	EPI_ISL_979735	MW701273

USA/CA-CZB-19142/2020	EPI_ISL_979736	MW701272
USA/CA-CZB-19143/2020	EPI_ISL_979737	MW701271
USA/CA-CZB-19144/2020	EPI_ISL_979738	MW701270
USA/CA-CZB-19145/2020	EPI_ISL_979739	MW701269
USA/CA-CZB-19146/2020	EPI_ISL_979740	MW701268
USA/CA-CZB-19147/2020	EPI_ISL_979741	MW701267
USA/CA-CZB-19148/2020	EPI_ISL_979742	MW701266
USA/CA-CZB-19149/2020	EPI_ISL_979743	MW701265
USA/CA-CZB-19150/2020	EPI_ISL_979744	MW701264
USA/CA-CZB-19151/2020	EPI_ISL_979745	MW701263
USA/CA-CZB-19152/2020	EPI_ISL_979746	MW701262
USA/CA-CZB-19153/2020	EPI_ISL_979747	MW701261
USA/CA-CZB-19154/2020	EPI_ISL_979748	MW701260
USA/CA-CZB-19155/2020	EPI_ISL_979749	MW701259
USA/CA-CZB-19156/2020	EPI_ISL_979750	MW701258
USA/CA-CZB-19157/2020	EPI_ISL_979751	MW701257
USA/CA-CZB-19158/2020	EPI_ISL_979752	MW701256
USA/CA-CZB-19159/2020	EPI_ISL_979753	MW701255
USA/CA-CZB-19160/2020	EPI_ISL_979754	MW701254
USA/CA-CZB-19161/2020	EPI_ISL_979755	MW701253
USA/CA-CZB-19162/2020	EPI_ISL_979756	MW701252
USA/CA-CZB-19163/2020	EPI_ISL_979757	MW701251
USA/CA-CZB-19164/2020	EPI_ISL_979758	MW701250
USA/CA-CZB-19165/2020	EPI_ISL_979759	MW701249
USA/CA-CZB-19166/2020	EPI_ISL_979760	MW701248
USA/CA-CZB-19167/2020	EPI_ISL_979761	MW701247
USA/CA-CZB-19168/2020	EPI_ISL_979762	MW701246
USA/CA-CZB-19169/2020	EPI_ISL_979763	MW701245
USA/CA-CZB-19170/2020	EPI_ISL_979764	MW701244

USA/CA-CZB-19171/2020	EPI_ISL_979765	MW701243
USA/CA-CZB-19172/2020	EPI_ISL_979766	MW701242
USA/CA-CZB-19173/2020	EPI_ISL_979767	MW701241
USA/CA-CZB-19174/2020	EPI_ISL_979768	MW701240
USA/CA-CZB-19175/2020	EPI_ISL_979769	MW701239
USA/CA-CZB-19176/2020	EPI_ISL_979770	MW701238
USA/CA-CZB-19177/2020	EPI_ISL_979771	MW701237
USA/CA-CZB-19178/2020	EPI_ISL_979772	MW701236
USA/CA-CZB-19179/2020	EPI_ISL_979773	MW701235
USA/CA-CZB-19180/2020	EPI_ISL_979774	MW701234
USA/CA-CZB-19181/2020	EPI_ISL_979775	MW701233
USA/CA-CZB-19182/2020	EPI_ISL_979776	MW701232
USA/CA-CZB-19183/2020	EPI_ISL_979777	MW701231
USA/CA-CZB-19184/2020	EPI_ISL_979778	MW701230
USA/CA-CZB-19185/2020	EPI_ISL_979779	MW701229
USA/CA-CZB-19186/2020	EPI_ISL_979780	MW701228
USA/CA-CZB-19187/2020	EPI_ISL_979781	MW701227
USA/CA-CZB-19188/2020	EPI_ISL_979782	MW701226
USA/CA-CZB-19189/2020	EPI_ISL_979783	MW701225
USA/CA-CZB-19190/2020	EPI_ISL_979784	MW701224
USA/CA-CZB-19191/2020	EPI_ISL_2758426	MZ842238
USA/CA-CZB-19192/2020	EPI_ISL_979785	MW701223
USA/CA-CZB-19193/2020	EPI_ISL_979786	MW701222
USA/CA-CZB-19194/2020	EPI_ISL_979787	MW701221
USA/CA-CZB-19195/2020	EPI_ISL_979788	MW701220
USA/CA-CZB-19196/2020	EPI_ISL_979789	MW701219
USA/CA-CZB-19197/2020	EPI_ISL_979790	MW701218
USA/CA-CZB-19199/2020	EPI_ISL_979791	MW701217
USA/CA-CZB-19200/2020	EPI_ISL_979792	MW701216

USA/CA-CZB-19201/2020	EPI_ISL_979793	MW701215
USA/CA-CZB-19202/2020	EPI_ISL_979794	MW701214
USA/CA-CZB-19203/2020	EPI_ISL_979795	MW701213
USA/CA-CZB-19204/2020	EPI_ISL_979796	MW701212
USA/CA-CZB-19205/2020	EPI_ISL_979797	MW701211
USA/CA-CZB-19207/2020	EPI_ISL_979162	MW700768
USA/CA-CZB-19208/2020	EPI_ISL_979163	MW700767
USA/CA-CZB-19209/2020	EPI_ISL_979164	MW700766
USA/CA-CZB-19210/2020	EPI_ISL_979165	MW700765
USA/CA-CZB-19211/2020	EPI_ISL_979166	MW700764
USA/CA-CZB-19212/2020	EPI_ISL_979167	MW700763
USA/CA-CZB-19214/2020	EPI_ISL_979168	MW700762
USA/CA-CZB-19215/2020	EPI_ISL_979169	MW700761
USA/CA-CZB-19216/2020	EPI_ISL_1030499	MW702982
USA/CA-CZB-19217/2020	EPI_ISL_979170	MW700760
USA/CA-CZB-19218/2020	EPI_ISL_979171	MW700759
USA/CA-CZB-19219/2020	EPI_ISL_979172	NA
USA/CA-CZB-19220/2020	EPI_ISL_979173	MW700758
USA/CA-CZB-19221/2020	EPI_ISL_979174	MW700757
USA/CA-CZB-19222/2020	EPI_ISL_979175	MW700756
USA/CA-CZB-19223/2020	EPI_ISL_979176	MW700755
USA/CA-CZB-19224/2020	EPI_ISL_979177	MW700754
USA/CA-CZB-19225/2020	EPI_ISL_979178	MW700753
USA/CA-CZB-19227/2020	EPI_ISL_979179	MW700752
USA/CA-CZB-19228/2020	EPI_ISL_979180	MW700751
USA/CA-CZB-19229/2020	EPI_ISL_979181	MW700750
USA/CA-CZB-19230/2020	EPI_ISL_979182	MW700749
USA/CA-CZB-19232/2020	EPI_ISL_979183	MW700748
USA/CA-CZB-19233/2020	EPI_ISL_979184	MW700747

USA/CA-CZB-19235/2020	EPI_ISL_979185	MW700746
USA/CA-CZB-19236/2020	EPI_ISL_979186	MW700745
USA/CA-CZB-19237/2020	EPI_ISL_979187	MW700744
USA/CA-CZB-19238/2020	EPI_ISL_979188	MW700743
USA/CA-CZB-19239/2020	EPI_ISL_979189	MW700742
USA/CA-CZB-19240/2020	EPI_ISL_979190	MW700741
USA/CA-CZB-19241/2020	EPI_ISL_979191	MW700740
USA/CA-CZB-19242/2020	EPI_ISL_979192	MW700739
USA/CA-CZB-19243/2020	EPI_ISL_979193	MW700738
USA/CA-CZB-19244/2020	EPI_ISL_979194	MW700737
USA/CA-CZB-19246/2020	EPI_ISL_979195	MW700736
USA/CA-CZB-19247/2020	EPI_ISL_979196	MW700735
USA/CA-CZB-19248/2020	EPI_ISL_979197	MW700734
USA/CA-CZB-19249/2020	EPI_ISL_979198	MW700733
USA/CA-CZB-19250/2020	EPI_ISL_979199	MW700732
USA/CA-CZB-19251/2020	EPI_ISL_979200	MW700731
USA/CA-CZB-19252/2020	EPI_ISL_979201	MW700730
USA/CA-CZB-19253/2020	EPI_ISL_979202	MW700729
USA/CA-CZB-19254/2020	EPI_ISL_979203	MW700728
USA/CA-CZB-19255/2020	EPI_ISL_979204	MW700727
USA/CA-CZB-19256/2020	EPI_ISL_979205	MW700726
USA/CA-CZB-19257/2020	EPI_ISL_979206	MW700725
USA/CA-CZB-19258/2020	EPI_ISL_979207	MZ842237
USA/CA-CZB-19259/2020	EPI_ISL_979208	MW700724
USA/CA-CZB-19261/2020	EPI_ISL_979209	MW700723
USA/CA-CZB-19262/2020	EPI_ISL_979210	MW700722
USA/CA-CZB-19263/2020	EPI_ISL_2658604	NA
USA/CA-CZB-19265/2020	EPI_ISL_979211	MW700721
USA/CA-CZB-19266/2020	EPI_ISL_979212	MW700720

USA/CA-CZB-19267/2020	EPI_ISL_979213	MW700719
USA/CA-CZB-19268/2020	EPI_ISL_979214	MW700718
USA/CA-CZB-19269/2020	EPI_ISL_979215	MW700717
USA/CA-CZB-19271/2020	EPI_ISL_979216	MW700716
USA/CA-CZB-19272/2020	EPI_ISL_979217	MW700715
USA/CA-CZB-19273/2020	EPI_ISL_979218	MW700714
USA/CA-CZB-19274/2020	EPI_ISL_979219	MW700713
USA/CA-CZB-19275/2020	EPI_ISL_979220	MW700712
USA/CA-CZB-19276/2020	EPI_ISL_979221	MW700711
USA/CA-CZB-19277/2020	EPI_ISL_979222	MW700710
USA/CA-CZB-19278/2020	EPI_ISL_979223	MW700709
USA/CA-CZB-19279/2020	EPI_ISL_979224	MW700708
USA/CA-CZB-19280/2020	EPI_ISL_979225	MW700707
USA/CA-CZB-19281/2020	EPI_ISL_979226	MW700706
USA/CA-CZB-19282/2020	EPI_ISL_979227	MW700705
USA/CA-CZB-19283/2020	EPI_ISL_979228	MW700704
USA/CA-CZB-19284/2020	EPI_ISL_979229	MW700703
USA/CA-CZB-19285/2020	EPI_ISL_979230	MW700702
USA/CA-CZB-19286/2020	EPI_ISL_979231	MW700701
USA/CA-CZB-19287/2020	EPI_ISL_979232	MZ842236
USA/CA-CZB-19288/2020	EPI_ISL_979233	MZ842235
USA/CA-CZB-19289/2020	EPI_ISL_979234	MW700700
USA/CA-CZB-19290/2020	EPI_ISL_979235	MW700699
USA/CA-CZB-19291/2020	EPI_ISL_979236	MW700698
USA/CA-CZB-19292/2020	EPI_ISL_979237	MW700697
USA/CA-CZB-19293/2020	EPI_ISL_979238	MW700696
USA/CA-CZB-19294/2020	EPI_ISL_979239	MW700695
USA/CA-CZB-19295/2020	EPI_ISL_979240	MW700694
USA/CA-CZB-19296/2020	EPI_ISL_979241	MW700693

USA/CA-CZB-19297/2020	EPI_ISL_979242	MW700692
USA/CA-CZB-19298/2020	EPI_ISL_979243	MW700691
USA/CA-CZB-19299/2020	EPI_ISL_979244	MW700690
USA/CA-CZB-19301/2020	EPI_ISL_979245	MW700689
USA/CA-CZB-19302/2020	EPI_ISL_955693	MW700467
USA/CA-CZB-19303/2020	EPI_ISL_2758427	MZ842234
USA/CA-CZB-19304/2020	EPI_ISL_955694	MW700466
USA/CA-CZB-19305/2020	EPI_ISL_955695	MW700465
USA/CA-CZB-19306/2020	EPI_ISL_955696	MW700464
USA/CA-CZB-19307/2020	EPI_ISL_955697	MW700463
USA/CA-CZB-19308/2020	EPI_ISL_955698	MW700462
USA/CA-CZB-19309/2020	EPI_ISL_955699	MW700461
USA/CA-CZB-19310/2020	EPI_ISL_955700	MW700460
USA/CA-CZB-19311/2020	EPI_ISL_955701	MW700459
USA/CA-CZB-19312/2020	EPI_ISL_955702	MW700458
USA/CA-CZB-19313/2020	EPI_ISL_955703	MW700457
USA/CA-CZB-19314/2020	EPI_ISL_955704	MW700456
USA/CA-CZB-19315/2020	EPI_ISL_955705	MW700455
USA/CA-CZB-19316/2020	EPI_ISL_955706	MW700454
USA/CA-CZB-19317/2020	EPI_ISL_955707	MW700453
USA/CA-CZB-19318/2020	EPI_ISL_955708	MW700452
USA/CA-CZB-19319/2020	EPI_ISL_955709	MW700451
USA/CA-CZB-19320/2020	EPI_ISL_955710	MW700450
USA/CA-CZB-19321/2020	EPI_ISL_955711	MW700449
USA/CA-CZB-19322/2020	EPI_ISL_955712	MW700448
USA/CA-CZB-19323/2020	EPI_ISL_955713	MW700447
USA/CA-CZB-19324/2020	EPI_ISL_955714	MW700446
USA/CA-CZB-19325/2020	EPI_ISL_955715	MW700445
USA/CA-CZB-19326/2020	EPI_ISL_955716	MW700444

USA/CA-CZB-19327/2020	EPI_ISL_955717	MW700443
USA/CA-CZB-19328/2020	EPI_ISL_955718	MW700442
USA/CA-CZB-19329/2020	EPI_ISL_955719	MW700441
USA/CA-CZB-19330/2020	EPI_ISL_955720	MW700440
USA/CA-CZB-19331/2020	EPI_ISL_955721	MW700439
USA/CA-CZB-19332/2020	EPI_ISL_955722	MW700438
USA/CA-CZB-19333/2020	EPI_ISL_955723	MW700437
USA/CA-CZB-19334/2020	EPI_ISL_1027561	MW703037
USA/CA-CZB-19335/2020	EPI_ISL_2659213	MW700436
USA/CA-CZB-19336/2020	EPI_ISL_955724	MW700435
USA/CA-CZB-19338/2020	EPI_ISL_955725	MW700434
USA/CA-CZB-19339/2020	EPI_ISL_955726	MW700433
USA/CA-CZB-19340/2020	EPI_ISL_955727	MW700432
USA/CA-CZB-19341/2020	EPI_ISL_2758428	MZ842233
USA/CA-CZB-19342/2020	EPI_ISL_955728	MW700431
USA/CA-CZB-19343/2020	EPI_ISL_955729	MW700430
USA/CA-CZB-19344/2020	EPI_ISL_955730	MW700429
USA/CA-CZB-19345/2020	EPI_ISL_2659635	MW700428
USA/CA-CZB-19346/2020	EPI_ISL_955731	MW700427
USA/CA-CZB-19347/2020	EPI_ISL_955732	MW700426
USA/CA-CZB-19348/2020	EPI_ISL_955733	MW700425
USA/CA-CZB-19349/2020	EPI_ISL_955734	MW700424
USA/CA-CZB-19350/2020	EPI_ISL_955735	MW700423
USA/CA-CZB-19351/2020	EPI_ISL_955736	MW700422
USA/CA-CZB-19352/2020	EPI_ISL_955737	MW700421
USA/CA-CZB-19353/2020	EPI_ISL_955738	MW700420
USA/CA-CZB-19354/2020	EPI_ISL_955739	MW700419
USA/CA-CZB-19355/2020	EPI_ISL_955740	MW700418
USA/CA-CZB-19356/2020	EPI_ISL_955741	MW700417

USA/CA-CZB-19357/2020	EPI_ISL_955742	MW700416
USA/CA-CZB-19358/2020	EPI_ISL_955743	MW700415
USA/CA-CZB-19359/2020	EPI_ISL_955744	MW700414
USA/CA-CZB-19360/2020	EPI_ISL_955745	MW700413
USA/CA-CZB-19361/2020	EPI_ISL_955746	MW700412
USA/CA-CZB-19362/2020	EPI_ISL_955747	MW700411
USA/CA-CZB-19363/2020	EPI_ISL_955748	MW700410
USA/CA-CZB-19364/2020	EPI_ISL_955749	MW700409
USA/CA-CZB-19365/2020	EPI_ISL_955750	MZ842232
USA/CA-CZB-19366/2020	EPI_ISL_955751	MW700408
USA/CA-CZB-19367/2020	EPI_ISL_955752	MW700407
USA/CA-CZB-19368/2020	EPI_ISL_955753	MW700406
USA/CA-CZB-19369/2020	EPI_ISL_2658710	NA
USA/CA-CZB-19371/2020	EPI_ISL_955754	MW700405
USA/CA-CZB-19372/2020	EPI_ISL_955755	MW700404
USA/CA-CZB-19373/2020	EPI_ISL_955756	MW700403
USA/CA-CZB-19374/2020	EPI_ISL_955757	MW700402
USA/CA-CZB-19375/2020	EPI_ISL_955758	MW700401
USA/CA-CZB-19376/2020	EPI_ISL_955759	MW700400
USA/CA-CZB-19377/2020	EPI_ISL_955760	MW700399
USA/CA-CZB-19378/2020	EPI_ISL_955761	MW700398
USA/CA-CZB-19379/2020	EPI_ISL_955762	MW700397
USA/CA-CZB-19380/2020	EPI_ISL_955763	MW700396
USA/CA-CZB-19381/2021	EPI_ISL_955764	MW700395
USA/CA-CZB-19382/2020	EPI_ISL_955765	MW700394
USA/CA-CZB-19383/2020	EPI_ISL_955766	MW700393
USA/CA-CZB-19384/2021	EPI_ISL_955767	MW700392
USA/CA-CZB-19385/2021	EPI_ISL_955768	MW700391
USA/CA-CZB-19387/2021	EPI_ISL_955769	MW700390

USA/CA-CZB-19388/2021	EPI_ISL_955770	MW700389
USA/CA-CZB-19389/2021	EPI_ISL_955771	MW700388
USA/CA-CZB-19390/2021	EPI_ISL_955772	MW700387
USA/CA-CZB-19391/2021	EPI_ISL_955773	MW700386
USA/CA-CZB-19392/2021	EPI_ISL_955774	MW700385
USA/CA-CZB-19393/2021	EPI_ISL_1027476	MW703036
USA/CA-CZB-19394/2021	EPI_ISL_955775	MW700384
USA/CA-CZB-19395/2021	EPI_ISL_955776	MW700383
USA/CA-CZB-19396/2021	EPI_ISL_1027517	MW703035
USA/CA-CZB-19397/2021	EPI_ISL_955777	MW700382
USA/CA-CZB-2033/2020	EPI_ISL_1924619	MZ842848
USA/CA-CZB-2034/2020	EPI_ISL_486280	MT750454
USA/CA-CZB-2035/2020	EPI_ISL_486281	MZ842847
USA/CA-CZB-2036/2020	EPI_ISL_486282	MT750455
USA/CA-CZB-2037/2020	EPI_ISL_486283	MT750456
USA/CA-CZB-2039/2020	EPI_ISL_486284	MZ842846
USA/CA-CZB-2041/2020	EPI_ISL_486285	MT750457
USA/CA-CZB-2042/2020	EPI_ISL_486286	MT750458
USA/CA-CZB-23111/2021	EPI_ISL_1185409	MW739359
USA/CA-CZB-23112/2021	EPI_ISL_1185355	MW739358
USA/CA-CZB-23113/2021	EPI_ISL_1185438	MW739357
USA/CA-CZB-23115/2021	EPI_ISL_1185428	MW739356
USA/CA-CZB-23116/2021	EPI_ISL_1185294	MW739355
USA/CA-CZB-23117/2021	EPI_ISL_1185302	MW739354
USA/CA-CZB-23118/2021	EPI_ISL_1185528	MW739353
USA/CA-CZB-23119/2021	EPI_ISL_1185359	MW739352
USA/CA-CZB-23120/2021	EPI_ISL_1185489	MW739351
USA/CA-CZB-23122/2021	EPI_ISL_1185361	MW739350
USA/CA-CZB-23123/2021	EPI_ISL_1185408	MW739349

USA/CA-CZB-23124/2021	EPI_ISL_1185497	MW739348
USA/CA-CZB-23125/2021	EPI_ISL_1185448	MW739347
USA/CA-CZB-23126/2021	EPI_ISL_1185505	MW739346
USA/CA-CZB-23127/2021	EPI_ISL_1185382	MW739345
USA/CA-CZB-23128/2021	EPI_ISL_1185385	MW739344
USA/CA-CZB-23129/2021	EPI_ISL_1185319	MW739343
USA/CA-CZB-23130/2021	EPI_ISL_1185318	MW739342
USA/CA-CZB-23131/2021	EPI_ISL_1185325	MW739341
USA/CA-CZB-23132/2021	EPI_ISL_1185395	MW739340
USA/CA-CZB-23133/2021	EPI_ISL_1185304	MW739339
USA/CA-CZB-23134/2021	EPI_ISL_1185345	MW739338
USA/CA-CZB-23135/2021	EPI_ISL_1185455	MW739337
USA/CA-CZB-23136/2021	EPI_ISL_1185352	MW739336
USA/CA-CZB-23137/2021	EPI_ISL_1185399	MW739335
USA/CA-CZB-23138/2021	EPI_ISL_1185298	MW739334
USA/CA-CZB-23139/2021	EPI_ISL_2659557	MW739333
USA/CA-CZB-23140/2021	EPI_ISL_1185460	MZ842204
USA/CA-CZB-23141/2021	EPI_ISL_1185369	MW739332
USA/CA-CZB-23142/2021	EPI_ISL_1185488	MW739331
USA/CA-CZB-23143/2021	EPI_ISL_1185326	MW739330
USA/CA-CZB-23144/2021	EPI_ISL_1185468	MW739329
USA/CA-CZB-23145/2021	EPI_ISL_2659338	MW739328
USA/CA-CZB-23146/2021	EPI_ISL_1185312	MW739327
USA/CA-CZB-23147/2021	EPI_ISL_1185471	MW739326
USA/CA-CZB-23148/2021	EPI_ISL_1185309	MW739325
USA/CA-CZB-23149/2021	EPI_ISL_1185300	MW739324
USA/CA-CZB-23150/2021	EPI_ISL_1185301	MW739323
USA/CA-CZB-23151/2021	EPI_ISL_1185292	MW739322
USA/CA-CZB-23152/2021	EPI_ISL_2659380	MW739321

USA/CA-CZB-23153/2021	EPI_ISL_2659359	MW739320
USA/CA-CZB-23155/2021	EPI_ISL_2659316	MW739319
USA/CA-CZB-23156/2021	EPI_ISL_1185474	MW739318
USA/CA-CZB-23157/2021	EPI_ISL_1185365	MW739317
USA/CA-CZB-23158/2021	EPI_ISL_1185454	MW739316
USA/CA-CZB-23159/2021	EPI_ISL_1185394	MW739315
USA/CA-CZB-23161/2021	EPI_ISL_1185509	MW739314
USA/CA-CZB-23162/2021	EPI_ISL_1185436	MW739313
USA/CA-CZB-23163/2021	EPI_ISL_1185407	MW739312
USA/CA-CZB-23164/2021	EPI_ISL_1185308	MW739311
USA/CA-CZB-23165/2021	EPI_ISL_1185346	MW739310
USA/CA-CZB-23166/2021	EPI_ISL_1185507	MW739309
USA/CA-CZB-23167/2021	EPI_ISL_1185417	MW739308
USA/CA-CZB-23168/2021	EPI_ISL_2659319	MW739307
USA/CA-CZB-23169/2021	EPI_ISL_2659336	MW739306
USA/CA-CZB-23170/2021	EPI_ISL_2659609	MW739305
USA/CA-CZB-23171/2021	EPI_ISL_1185526	MW739304
USA/CA-CZB-23172/2021	EPI_ISL_1185360	MW739303
USA/CA-CZB-23173/2021	EPI_ISL_1185287	MW739302
USA/CA-CZB-23174/2021	EPI_ISL_1185476	MW739301
USA/CA-CZB-23175/2021	EPI_ISL_1185440	MW739300
USA/CA-CZB-23177/2021	EPI_ISL_1185329	MW739299
USA/CA-CZB-23178/2021	EPI_ISL_1185424	MW739298
USA/CA-CZB-23179/2021	EPI_ISL_1185291	MW739297
USA/CA-CZB-23180/2021	EPI_ISL_1185479	MW739296
USA/CA-CZB-23181/2021	EPI_ISL_1185481	MW739295
USA/CA-CZB-23182/2021	EPI_ISL_1185469	MW739294
USA/CA-CZB-23183/2021	EPI_ISL_1185323	MW739293
USA/CA-CZB-23184/2021	EPI_ISL_1185353	MW739292

USA/CA-CZB-23185/2021	EPI_ISL_1185519	MW739291
USA/CA-CZB-23186/2021	EPI_ISL_1185521	MW739290
USA/CA-CZB-23187/2021	EPI_ISL_1185328	MW739289
USA/CA-CZB-23188/2021	EPI_ISL_1185504	MW739288
USA/CA-CZB-23189/2021	EPI_ISL_1185413	MW739287
USA/CA-CZB-23190/2021	EPI_ISL_1185410	MW739286
USA/CA-CZB-23191/2021	EPI_ISL_2659421	MW739285
USA/CA-CZB-23192/2021	EPI_ISL_1185340	MW739284
USA/CA-CZB-23193/2021	EPI_ISL_1185498	MW739283
USA/CA-CZB-23194/2021	EPI_ISL_1185425	MW739282
USA/CA-CZB-23195/2021	EPI_ISL_1185324	MZ842203
USA/CA-CZB-23196/2021	EPI_ISL_1185516	MW739281
USA/CA-CZB-23198/2021	EPI_ISL_1185432	MW739280
USA/CA-CZB-23199/2021	EPI_ISL_1185518	MW739279
USA/CA-CZB-23200/2021	EPI_ISL_1185486	MW739278
USA/CA-CZB-23201/2021	EPI_ISL_1185487	MW739277
USA/CA-CZB-23202/2021	EPI_ISL_1185463	MW739276
USA/CA-CZB-23203/2021	EPI_ISL_1185306	MW739275
USA/CA-CZB-23204/2021	EPI_ISL_1185439	MW739274
USA/CA-CZB-23205/2021	EPI_ISL_2659265	MW739273
USA/CA-CZB-23206/2021	EPI_ISL_1185412	MW739272
USA/CA-CZB-2419/2020	EPI_ISL_513840	MZ842842
USA/CA-CZB-2420/2020	EPI_ISL_513841	MW035992
USA/CA-CZB-2421/2020	EPI_ISL_513842	MW035999
USA/CA-CZB-2422/2020	EPI_ISL_513843	MW036022
USA/CA-CZB-2423/2020	EPI_ISL_513844	MW036010
USA/CA-CZB-2424/2020	EPI_ISL_513845	MW035997
USA/CA-CZB-2425/2020	EPI_ISL_513846	MW036024
USA/CA-CZB-2426/2020	EPI_ISL_513847	MW036047

USA/CA-CZB-2429/2020	EPI_ISL_513848	MW036038
USA/CA-CZB-2430/2020	EPI_ISL_513849	MW035991
USA/CA-CZB-2431/2020	EPI_ISL_513850	MW036084
USA/CA-CZB-2433/2020	EPI_ISL_513851	NA
USA/CA-CZB-2436/2020	EPI_ISL_513852	MW036056
USA/CA-CZB-2443/2020	EPI_ISL_513853	MW036090
USA/CA-CZB-2445/2020	EPI_ISL_513854	MW036003
USA/CA-CZB-2446/2020	EPI_ISL_513855	MW036073
USA/CA-CZB-25659/2021	EPI_ISL_1234789	MW739803
USA/CA-CZB-25660/2021	EPI_ISL_1234794	MW739802
USA/CA-CZB-25661/2021	EPI_ISL_1234799	MW739801
USA/CA-CZB-25662/2021	EPI_ISL_1235011	MW739800
USA/CA-CZB-25663/2021	EPI_ISL_1234833	MW739799
USA/CA-CZB-25664/2021	EPI_ISL_1234871	MW739798
USA/CA-CZB-25665/2021	EPI_ISL_2658600	NA
USA/CA-CZB-25666/2021	EPI_ISL_1234807	MW739797
USA/CA-CZB-25667/2021	EPI_ISL_1234930	MW739796
USA/CA-CZB-25668/2021	EPI_ISL_1234826	MW739795
USA/CA-CZB-25669/2021	EPI_ISL_1234831	MW739794
USA/CA-CZB-25670/2021	EPI_ISL_1234793	MW739793
USA/CA-CZB-25671/2021	EPI_ISL_1235012	MW739792
USA/CA-CZB-25672/2021	EPI_ISL_1235023	MW739791
USA/CA-CZB-25674/2021	EPI_ISL_1234955	MW739790
USA/CA-CZB-25675/2021	EPI_ISL_1234949	MW739789
USA/CA-CZB-25676/2021	EPI_ISL_1234836	MW739788
USA/CA-CZB-25678/2021	EPI_ISL_1235002	MW739787
USA/CA-CZB-25679/2021	EPI_ISL_1234908	MW739786
USA/CA-CZB-25680/2021	EPI_ISL_1235030	MW739785
USA/CA-CZB-25681/2021	EPI_ISL_1235046	MW739784

USA/CA-CZB-25682/2021	EPI_ISL_1234788	MW739783
USA/CA-CZB-25683/2021	EPI_ISL_1235004	MW739782
USA/CA-CZB-25684/2021	EPI_ISL_2658582	NA
USA/CA-CZB-25685/2021	EPI_ISL_1234896	MW739781
USA/CA-CZB-25686/2021	EPI_ISL_1234898	MW739780
USA/CA-CZB-25687/2021	EPI_ISL_1234922	MW739779
USA/CA-CZB-25688/2021	EPI_ISL_1234852	MW739778
USA/CA-CZB-25689/2021	EPI_ISL_1235039	MW739777
USA/CA-CZB-25690/2021	EPI_ISL_1234927	MW739776
USA/CA-CZB-25691/2021	EPI_ISL_1235037	MW739775
USA/CA-CZB-25692/2021	EPI_ISL_1235003	MW739774
USA/CA-CZB-25693/2021	EPI_ISL_1234859	MW739773
USA/CA-CZB-25694/2021	EPI_ISL_1234998	MW739772
USA/CA-CZB-25695/2021	EPI_ISL_1234948	MW739771
USA/CA-CZB-25696/2021	EPI_ISL_1234889	MW739770
USA/CA-CZB-25697/2021	EPI_ISL_1234823	MW739769
USA/CA-CZB-25698/2021	EPI_ISL_1234981	MW739768
USA/CA-CZB-25699/2021	EPI_ISL_1234960	MW739767
USA/CA-CZB-25701/2021	EPI_ISL_1234882	MW739766
USA/CA-CZB-25702/2021	EPI_ISL_1234868	MW739765
USA/CA-CZB-25703/2021	EPI_ISL_1234989	MW739764
USA/CA-CZB-25704/2021	EPI_ISL_1235001	MW739763
USA/CA-CZB-25707/2021	EPI_ISL_1235005	MW739762
USA/CA-CZB-25708/2021	EPI_ISL_2758452	MZ842102
USA/CA-CZB-25709/2021	EPI_ISL_1235040	MW739761
USA/CA-CZB-25710/2021	EPI_ISL_1234824	MW739760
USA/CA-CZB-25711/2021	EPI_ISL_1234861	MW739759
USA/CA-CZB-25712/2021	EPI_ISL_1234971	MW739758
USA/CA-CZB-25713/2021	EPI_ISL_1234801	MW739757

USA/CA-CZB-25714/2021	EPI_ISL_1234903	MW739756
USA/CA-CZB-25715/2021	EPI_ISL_1234910	MW739755
USA/CA-CZB-25716/2021	EPI_ISL_1234853	MW739754
USA/CA-CZB-25718/2021	EPI_ISL_1234811	MW739753
USA/CA-CZB-25719/2021	EPI_ISL_1234934	MW739752
USA/CA-CZB-25720/2021	EPI_ISL_1234912	MW739751
USA/CA-CZB-25721/2021	EPI_ISL_1234814	MW739750
USA/CA-CZB-25722/2021	EPI_ISL_1235033	MW739749
USA/CA-CZB-25723/2021	EPI_ISL_1234911	MW739748
USA/CA-CZB-25724/2021	EPI_ISL_1234803	MW739747
USA/CA-CZB-25725/2021	EPI_ISL_1234874	MW739746
USA/CA-CZB-25726/2021	EPI_ISL_2658646	NA
USA/CA-CZB-25727/2021	EPI_ISL_1234936	MW739745
USA/CA-CZB-25728/2021	EPI_ISL_1234916	MW739744
USA/CA-CZB-25729/2021	EPI_ISL_1234822	MW739743
USA/CA-CZB-25730/2021	EPI_ISL_1234980	MW739742
USA/CA-CZB-25731/2021	EPI_ISL_1234805	MW739741
USA/CA-CZB-25732/2021	EPI_ISL_1234786	MW739740
USA/CA-CZB-25733/2021	EPI_ISL_1234817	MW739739
USA/CA-CZB-25734/2021	EPI_ISL_1234906	MW739738
USA/CA-CZB-25735/2021	EPI_ISL_1234816	MW739737
USA/CA-CZB-25736/2021	EPI_ISL_1234863	MW739736
USA/CA-CZB-25737/2021	EPI_ISL_1234890	MW739735
USA/CA-CZB-25738/2021	EPI_ISL_1235045	MW739734
USA/CA-CZB-25739/2021	EPI_ISL_1234821	MW739733
USA/CA-CZB-25740/2021	EPI_ISL_1234931	MW739732
USA/CA-CZB-25741/2021	EPI_ISL_1235053	MW739731
USA/CA-CZB-25742/2021	EPI_ISL_1234928	MW739730
USA/CA-CZB-25743/2021	EPI_ISL_1234888	MW739729

USA/CA-CZB-25744/2021	EPI_ISL_1234893	MW739728
USA/CA-CZB-25745/2021	EPI_ISL_1234984	MW739727
USA/CA-CZB-25746/2021	EPI_ISL_1234914	MW739726
USA/CA-CZB-25747/2021	EPI_ISL_1234905	MW739725
USA/CA-CZB-25748/2021	EPI_ISL_2758453	MZ842101
USA/CA-CZB-25749/2021	EPI_ISL_1234792	MW739724
USA/CA-CZB-25750/2021	EPI_ISL_1234966	MW739723
USA/CA-CZB-25751/2021	EPI_ISL_1234963	MW739722
USA/CA-CZB-25752/2021	EPI_ISL_1234856	MW739721
USA/CA-CZB-25753/2021	EPI_ISL_2659310	MZ842100
USA/CA-CZB-25754/2021	EPI_ISL_1234974	MW739720
